# *Mycobacterium tuberculosis* manipulates LINC02528 in macrophages to modulate anti-tuberculosis metabolic immunity

**DOI:** 10.1371/journal.ppat.1013810

**Published:** 2025-12-23

**Authors:** Yuzhong Xu, Kehong Zhang, Sinan Li, Lin Qiao, Siwei Mo, Wenfei Wang, Jialou Zhu, Xiaoqian Liu, Ningjian Cai, Chenyan Shi, Yi Cai, Yunlong Hu, Xinchun Chen

**Affiliations:** 1 Department of Clinical Laboratory, Shenzhen Baoan Hospital/The Second Affiliated Hospital of Shenzhen University, Shenzhen University Medical School, Shenzhen, China; 2 Guangdong Provincial Key Laboratory of Infection Immunity and Inflammation, Department of Pathogen Biology, Shenzhen University Medical School, Shenzhen, China; 3 Guangdong Provincial Clinical Research Center for Laboratory Medicine, Guangzhou, China; 4 College of biological sciences, China Agricultural University, Beijing, China; 5 Department of Laboratory Medicine, Affiliated Hospital of Zunyi Medical University/Zunyi Medical University, Zunyi, China; 6 National Clinical Research Center for Infectious Diseases, Shenzhen Third People’s Hospital, The Second Affiliated Hospital, School of Medicine, Southern University of Science and Technology, Shenzhen, China; 7 Department of Clinical Laboratory, Guangzhou Chest Hospital, Guangzhou/State Key Laboratory of Respiratory Disease, Guangzhou, China; 8 Department of Life Science and Technology, Wuhan Polytechnic University, Wuhan, China; 9 National Clinical Laboratory on Tuberculosis, Beijing Key Laboratory for Drug-Resistant Tuberculosis Research, Beijing Chest Hospital, Capital Medical University, Beijing Tuberculosis and Thoracic Tumor Institute, Beijing, China; New Jersey Medical School, UNITED STATES OF AMERICA

## Abstract

Host defenses are crucial in deciding the fate of *Mycobacterium tuberculosis* (*Mtb*) infections, as less than 10% of infected individuals develop tuberculosis. Oxidative stress plays a critical role in the host defense against *Mtb*. However, the mechanisms by which *Mtb* modulates redox homeostasis to evade immune responses remain poorly understood. In this study, we primarily identified a pathogen-responsive long noncoding RNA, LINC02528, which was selectively upregulated in peripheral blood mononuclear cells (PBMCs) from tuberculosis (TB) patients. In *Mtb*-infected macrophages, LINC02528 dynamically relocalizes from the nucleus to the cytoplasm. Functionally, CRISPR-Cas9-mediated knockout (KO) of LINC02528 in macrophages resulted in reduced *Mtb* survival concurrent with an elevated IL-1β expression. Importantly, these antimicrobial effects were abrogated by IL-1 receptor antagonist (IL-RA) treatment. Interestingly, LINC02528 was found to directly bind to TOMM22, a mitochondrial outer membrane translocase, as validated by co-localization analysis using in situ hybridization of lung tissue sections from a TB patient. The ECAR results revealed that LINC02528 deficiency significantly increased glycolysis and elevated *Mtb*-induced mitochondrial ROS (mtROS) production. Notably, TOMM22 knockdown phenocopied LINC02528 deletion effects, suggesting functional interdependence in modulating mitochondrial dynamics and the host’s anti-TB immunity. Collectively, our findings reveal a novel strategy wherein *Mtb* hijacks the lncRNA-mitochondrial axis to rewire redox-metabolic checkpoints to favor immune evasion. Targeting LINC02528 could dually disrupt the pathogen-permissive redox balance and activate mtROS-IL-1β-mediated antimicrobial defense, offering novel therapeutic avenues for TB.

## Author summary

Tuberculosis (TB), caused by *Mycobacterium tuberculosis* (*Mtb*), relies on host immune evasion for survival. While oxidative stress is critical for defense, how *Mtb* disrupts redox balance remains unclear. We discovered a long noncoding RNA, LINC02528, upregulated in TB patients’ immune cells, which relocates during infection. Deleting LINC02528 in macrophages reduced bacterial survival and boosted IL-1β, a key antimicrobial signal. Mechanistically, LINC02528 binds TOMM22, a mitochondrial protein, to suppress glycolysis and reactive oxygen species (ROS)—helping *Mtb* evade immunity. Strikingly, blocking TOMM22 mimicked LINC02528 loss, confirming their shared role in metabolic hijacking. Our work reveals how *Mtb* exploits the LINC02528-TOMM22 axis to weaken host defenses. Targeting this pathway could starve *Mtb* of its redox shelter while activating IL-1β-driven immunity, offering a dual-action strategy against TB.

## Introduction

Tuberculosis (TB), caused by an infection of *Mycobacterium tuberculosis* (*Mtb*), remains a leading infectious disease worldwide that kills over 1.2 million people per year [1]. While infection with *Mtb* is essential for TB, the outcome of an *Mtb* infection is more likely to be determined by the effectiveness of host defenses against *Mtb*, as nearly 90% of infected subjects do not become active TB cases [2]. Macrophages play a central role in either providing a niche for bacterial replication or effectively killing bacteria [3]. Therefore, appropriate macrophage activation—achieving a balanced response that effectively controls bacterial growth while minimizing host tissue damage—is a key determinant of the outcome of *Mtb* infection [4,5]. However, how macrophages control and eliminate *Mtb* and how *Mtb* manipulates macrophage cell machinery to create a permissive niche remains to be elucidated.

Long non-coding RNAs (lncRNAs), usually defined as transcripts that are longer than 200 nucleotides but lack protein-coding capacity, have attracted much attention from researchers due to their implicitly important biological functions. A small fraction of lncRNAs explored have demonstrated a strong association with innate immunity [6,7]. Multiple lncRNAs appear to be markedly dysregulated in TB, an effect that is sometimes accompanied by a pattern of tissue-specific expression that facilitates the use of lncRNAs for diagnostic, prognostic, and even therapeutic purposes [8]. Many studies have identified a number of significantly differentially expressed lncRNAs in peripheral blood mononuclear cells (PBMCs) from TB patients and have aimed to select the most indicative lncRNAs as biomarkers for active pulmonary tuberculosis [9,10]. LncRNAs are involved in the innate immune activity regulated by macrophages, and inducible inflammatory gene expression patterns are critical for human defenses against microbes [11,12]. For instance, to control inflammatory responses to *Mtb* infection, lncRNA-Cox2 regulates the activation or repression of immune-related genes that activate NF-κB and STAT signaling pathways [13,14]. In addition, the status quo is that most human lncRNAs are not conserved in mice, and modern biological approaches used in elucidating the roles of noncoding RNAs have had limited success, both of which add to the difficulty of exploring these lncRNAs.

In this study, we used whole-transcriptome sequencing (RNA-seq) of TB patient and healthy donor PBMCs to annotate abnormally expressed macrophage lncRNAs. Those lncRNAs highly expressed in the TB group were then prioritized for further study. We identified a functional lncRNA (LINC02528) in TB samples that has been recently annotated as a suppressor of inflammatory macrophage-specific lncRNA in M1 type inflammatory macrophages in human atherosclerosis by inducing apoptosis [15]. However, it did not induce apoptosis in our cell model (possibly due to differences in experimental design, time window of infection, and purpose), and the mechanisms of its effect during *Mtb* infection were not clear. The interaction between LINC02528 and TOMM22 suppressed mtROS production, which in turn reduced IL-1β secretion and promoted intracellular bacterial growth. Previous studies have documented that during TB infection, host cells undergo profound metabolic reprogramming, leading to differential regulation of various cytokines and chemokines associated with inflammation, clearance, inhibition, and progression of *Mtb* infection [16,17]. We therefore hypothesize that this lncRNA regulates mitochondria metabolic activity and inflammation to maintain homeostasis in *Mtb*-infected macrophages.

## Results

### LINC02528 is notably upregulated in macrophages from TB patients

**Fig 1:**
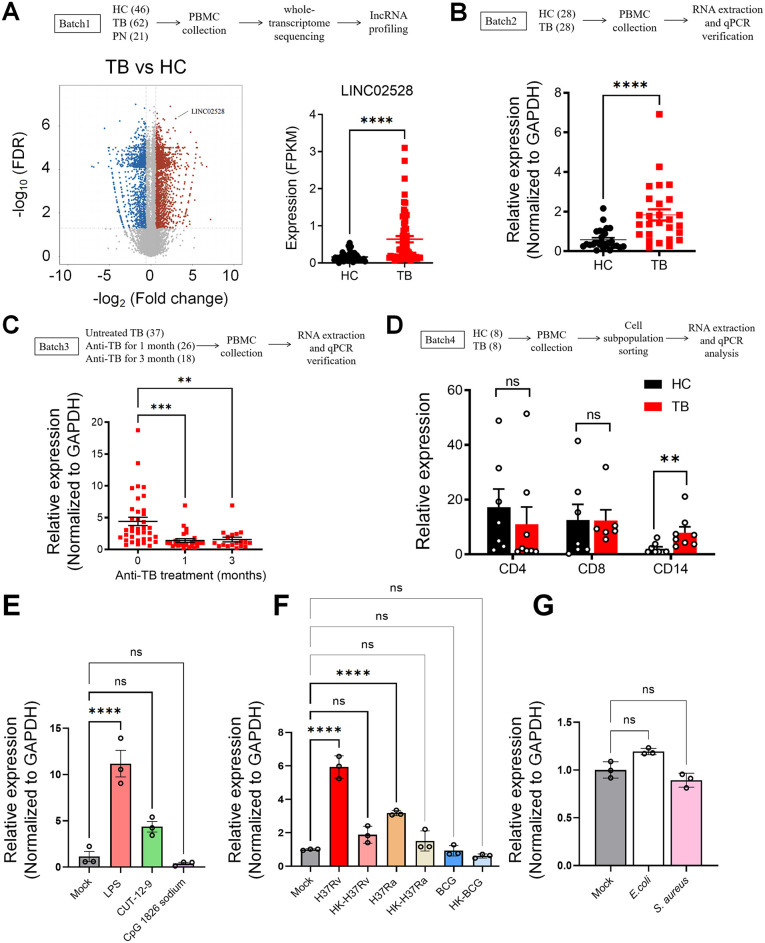
Characterization of LINC02528, a long noncoding RNA elevated in TB patients. **(A)** (Left) Volcano plots showing differentially expressed IncRNAs in RNA seq data for PBMCs from TB patients (n = 62) and healthy individuals (n = 46); (Right) FPKM expression of LINC02528 in TB compared to HC group; **(B)** LINC02528 mRNA expression level in another new batch of TB (n = 28) and healthy groups (n = 28). **(C)** Changes in LINC02528 expression after TB treatment: 0, active TB (n = 37); 1, after treatment for 1 month (n = 26); 3, after treatment for 3 months (n = 18). **(D)** Cell sub-populations (CD4, CD8, CD14) were sorted by FCM from TB patients (n = 8) and HC people (n = 8), followed by RNA extraction and cDNA synthesis, and the expression of LINC02528 was detected in the cDNA samples. **(E)** LINC02528 gene expression stimulated by LPS (100 ng/mL), CUT-12-9 (10 μM), and CpG1826 sodium (1 μM). **(F)** LINC02528 gene expression in heat treatment-killed or unkilled H37Rv, H37Ra and BCG-infected macrophages. **(G)** LINC02528 gene expression in untreated, *E. coli* (MOI = 10, 4 h) and *S. aureus* (MOI = 1, 4 h) -infected macrophages. The data represent the mean ± SEM. Unpaired 2-tailed student t test or one-way ANOVA Dunnett’s multiple comparisons test was used. Not significant (ns), *p < 0.05, ** *p *< 0.01, *** *p* < 0.005, **** *p* < 0.001.

Using PBMC samples collected from three distinct cohorts (Batch 1: n = 62 TB patients, n = 26 pneumonia (PN) patients, and n = 46 healthy controls (HC), we conducted whole-transcriptome sequencing and comprehensive lncRNA expression profiling to identify TB-specific lncRNAs implicated in host-pathogen interactions. Through systematic filtering using stringent screening criteria (TB-vs-PN and HC: 3442 differentially expressed lncRNAs, Q-value≤0.05 and |log_2_Ratio| >= 0.58496 (Fold change = 1.5)), we identified 318 differentially expressed lncRNAs among the TB, PN and HC groups, after filtering out transcripts with predicted protein-coding potential (S1A Fig). Notably, LINC02528 emerged as one of the most significantly upregulated lncRNAs in TB patients, and FPKM (Fragments Per Kilobase of exon per Million fragments mapped) was significantly higher in the TB group than in the HC group (Fig 1A, *p* < 0.001). Comparative analysis demonstrated that LINC02528 expression was significantly higher in TB patients compared to PN cases (S1B Fig, *p* < 0.05), indicating its specificity for TB pathogenesis and potential utility as a biomarker. In a comprehensive whole transcriptomic sequencing study, differential expression analysis (with thresholds set at |Fold change| ≥ 1.5, Q-value ≤ 0.05, and FPKM > 0.5 in at least one sample, excluding BGI genes) identified 697 differentially expressed lncRNAs and 3,132 differentially expressed mRNAs. To investigate potential cis-regulatory relationships, protein-coding genes located within 10 kb upstream or downstream of these lncRNAs were examined, revealing 642 significant lncRNA-mRNA cis-acting pairs. Subsequent Reactome pathway analysis was performed on these correlated mRNAs using overrepresentation analysis (e.g., Fisher’s exact test with FDR correction), which identified significantly enriched pathways—such as metabolic processes or signal transduction—to infer the functional roles of the lncRNAs in the biological context of interest. Reactome enrichment analysis of the expression profiles of mRNAs correlated with LINC02528 revealed significant enrichment in immune-related pathways and cytokine signaling (S1C Fig), thereby providing additional evidence for its potential involvement in host defense mechanisms. In fact, as shown in S1D Fig, we screened the top 4 lncRNAs from the batch 1 (excluding lncRNAs not registered in NCBI/Ensemble) and presented their FPKM values. Screening of candidate lncRNAs (SiFAM225A and SiLINC02555) revealed no significant impact on bacterial growth in the *Mtb*-infected macrophage model (S1E Fig). In contrast, LINC02528 knockdown demonstrated a statistically significant reduction in CFU counts, indicating its specific and potent role in host-pathogen interactions. Consequently, LINC02528 was selected for further investigation as the primary focus of this study. Quantitative PCR (qPCR) analysis using an independent validation cohort (Batch 2: n = 28 TB and n = 28 HC) confirmed the significantly elevated expression of LINC02528 in TB patients (Fig 1B). To further explore its clinical relevance, we investigated the dynamic changes in LINC02528 expression during anti-TB therapy using a longitudinal cohort (Batch 3: n = 37, untreated TB patients; n = 26, patients treated for one-month; and n = 18, patients treated for three-month). Notably, we observed a progressive decrease in LINC02528 expression levels in TB patients after treatment with anti-tuberculosis drugs, but it did not maintain a positive correlation with treatment duration (Fig 1C), which overall could indicate its potential role in monitoring treatment response. To identify the cellular source of LINC02528, we performed fluorescence-activated cell sorting (FACS) of PBMC subpopulations from an additional cohort (Batch 4: n = 8 TB patients and n = 8 HC), followed by qPCR analysis. While detectable across all cell types, LINC02528 expression was significantly enriched in CD14+ monocytes from TB patients compared to healthy controls (Fig 1D). By adding different stimuli such as LPS, CUT-12–9 and CPG as TLR2,4 and 9 agonists in THP-1 cells, only LPS significantly increased the expression of LINC02528 (Fig 1E). When treated with CUT-12–9, the cells showed a significant upregulation of LINC02528 mRNA expression compared to the untreated control group by unpaired t-test. A substantial body of literature supports a critical and central role for TLR2 and TLR4 in recognizing *Mtb* and initiating an inflammatory response [18]. This is also consistent with the data from the Muredach P Reilly’ group that LPS induction of M1 macrophages induces LINC02528 expression [15]. Next, we questioned whether strains and virulence factors could affect LINC02528 expression in THP-1 cells, we found that heat-killed H37Ra could not induce the expression of LINC02528 like normal H37Ra, whereas BCG did not affect the expression results regardless of heat-killed or non-heat-killed (Fig 1F). *E. coli* and *S. aureus*-infected macrophages did not up-regulate LINC02528 expression (Fig 1G), suggesting that it has a tuberculosis-specific role. This further demonstrates the importance of strain specificity and virulence factors. Based on the specific expression profile of LINC02528 in TB and its significant association with immune signaling pathways, we focused on elucidating the biological functions of this lncRNA and its molecular mechanisms in regulating host-pathogen immune interactions. Given that macrophages function as the primary defense mechanism against *Mtb* infection in vivo and considering the species-specific expression of LINC02528 in humans but not mice, we proceeded with subsequent studies using human cells, including primary human macrophages (human monocyte-derived macrophages, hMDMs) and the well-established THP-1 cell line for macrophages differentiation.

### *M. tuberculosis* infection induces the upregulation of LINC02528 expression and its cytoplasmic translocation to facilitate *Mtb* growth within macrophages

**Fig 2:**
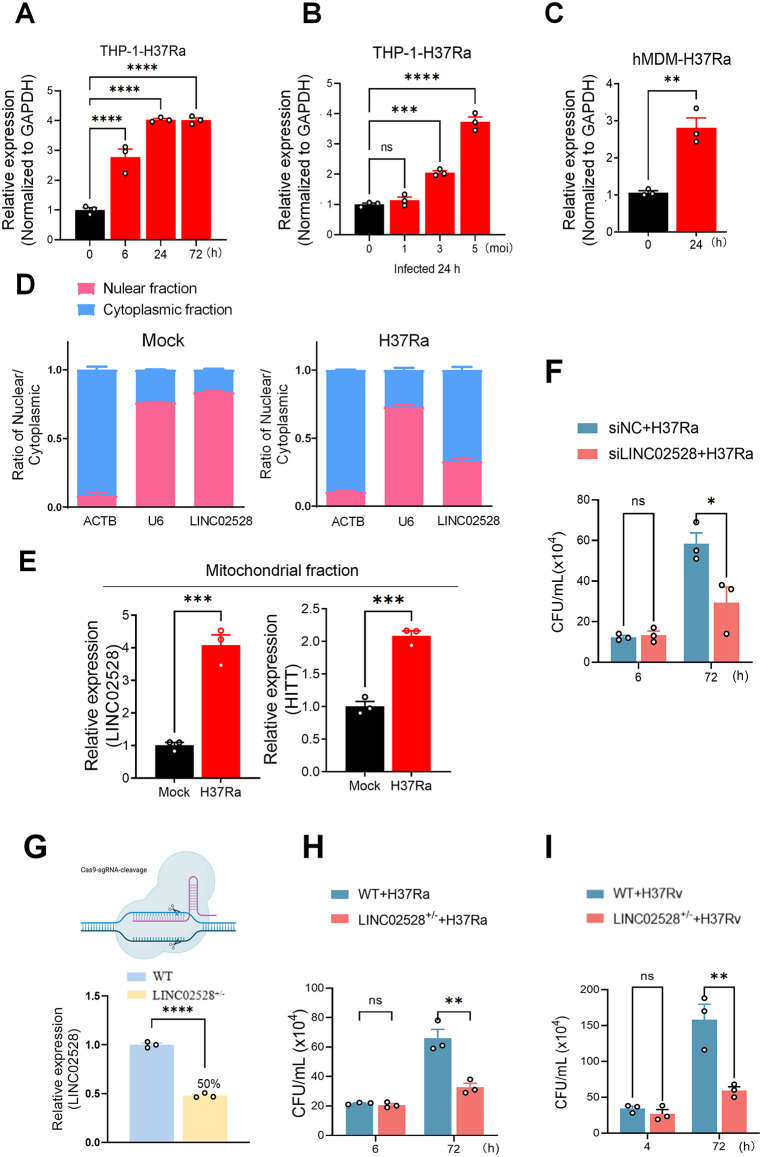
*M. tuberculosis* infection increased LINC02528 expression and translocated it to cytoplasm. **(A)** Time-dependent expression of LINC02528 in THP-1 cells infected with *Mtb* H37Ra at an MOI of 5. **(B)** Dose-dependent expression of LINC02528 in THP-1 cells 24 hours post-infection with *Mtb* H37Ra at varying MOIs. **(C)** LINC02528 expression in human monocyte-derived macrophages (hMDMs) infected with *Mtb*. **(D)** qRT-PCR analysis from isolated nuclear and cytoplasmic fraction in uninfected and *Mtb*-infected THP-1 cells, ACTB as cytoplasmic marker, U6 indicated nuclear located. **(E)** qRT-PCR analysis from isolated mitochondrial fraction in uninfected and *Mtb*-infected THP-1 cells, HITT is a reported mitochondrial lncRNA. **(F)** Intracellular *Mtb* colony forming units (CFU) of LINC02528-deficient macrophages mediated by SiRNA infected with H37Ra (MOI = 5) for 6 and 72 h. **(G)** Representative diagram of CRISPR-Cas9-based (This figure was generated using BioRender (https://biorender.com/)) LINC02528 knockout in THP-1 cell line, and histogram showing reduction in LINC02528 expression after using CRISPR-Cas9. **(H)** CFU of LINC02528^+/-^ macrophages infected with H37Ra (MOI = 5) for 6 and 72 h. **(I)** CFU of LINC02528^+/-^ macrophages infected with H37Rv (MOI = 5) for 4 and 72 h. The data represent the mean ± SEM from 3 independent experiments. Unpaired 2-tailed student t test or One-way ANOVA Dunnett’s/Sidak’s multiple comparisons test was used. Not significant (ns), *p < 0.05, ** *p* < 0.01, *** *p* < 0.005, **** *p* < 0.001.

Using a differentiated THP-1 macrophage cell line, we found that LINC02528 expression progressively increased in a time- and concentration (MOI)-dependent manner following infection with the *Mtb* H37Ra strain (Fig 2A and 2B). Furthermore, an infection duration of 24 hours with an MOI of 5 was identified to be the optimal conditions for subsequent cell infection experiments. Primary hMDMs were subsequently used to validate the significant upregulation of LINC02528 expression in macrophages induced by H37Ra infection (Fig 2C). A comparable upregulation of LINC02528 was also observed upon infection with the virulent H37Rv strain (S2A and S2B Fig). We analyzed LINC02528 expression via qPCR in a cohort comprising healthy controls (HC, n = 29), LTBI individuals (n = 22), and active TB patients (n = 28) (refer to the Batch 5 schematic for detailed cohort information). Our findings revealed no difference in LINC02528 expression in the LTBI group compared to the HC group (S3A Fig). Nucleus-cytoplasm separation in THP-1 cells followed by qPCR analysis revealed that LINC0258 was predominantly expressed in the nucleus under resting conditions but appeared to translocated to the cytoplasm upon *Mtb* infection (ACTB for cytoplasm, U6 for nucleus) (Fig 2D). Immunoblotting analysis confirmed the purity of subcellular fraction of LaminB1 in nuclear extract and β-Actin in cytoplasmic extract (S3B Fig). Mitochondrial fraction analysis in THP-1 cells revealed an approximately 3-fold upregulation of the lncRNA LINC02528 in infected macrophages compared to uninfected controls, implicating its role in the mitochondrial stress response to infection (Fig 2E). Additionally, HITT, a known mitochondrial-localized lncRNA [19], is also upregulated in infected macrophages, reinforcing the notion that *Mtb* infection alters the expression of mitochondrial lncRNAs. These findings highlight the dynamic regulation of mitochondrial-associated transcripts in macrophages during *Mtb* infection, possibly contributing to host-pathogen interactions and metabolic reprogramming. Immunoblotting analysis confirmed the selective enrichment of VDAC1 in mitochondrial fractions and GAPDH in cytosolic fractions (S3C Fig). To investigate the function of LINC02528, we first employed siRNA knockdown technology and the results demonstrated that knockdown of LINC02528 significantly decreased the intracellular load of *Mtb* (Fig 2F), and the detail information of siLINC02528 is shown in S2C Fig. Additionally, we performed gene editing on a THP-1 cell line using CRISPR-Cas9 technology, its characteristics were detailed in Figs 2G and S4A-C. Given that homozygous deletion of LINC02528 (LINC02528^-/-^) is lethal to cells, we opted for a heterozygous knockout strategy (denoted as “KO/+” or “+/-”), wherein only one allele of the target gene is modified or disrupted while the other remains functional. Consequently, we used this heterozygous LINC02528+ ^/-^ THP-1 cell line to examine the effects of partial loss of gene function (with approximately 50% reduction in gene expression) (Fig 2G). Colony-forming unit (CFU) assays revealed that LINC02528+ ^/-^ macrophages did not affect bacterial phagocytosis at 6 h post-infection, but led to substantial growth inhibition of H37Ra (Fig 2H) and H37Rv (Fig 2I) at 72 h post-infection.

### LINC02528 knockdown suppressed intracellular *Mtb* growth by enhancing IL-1β production

**Fig 3:**
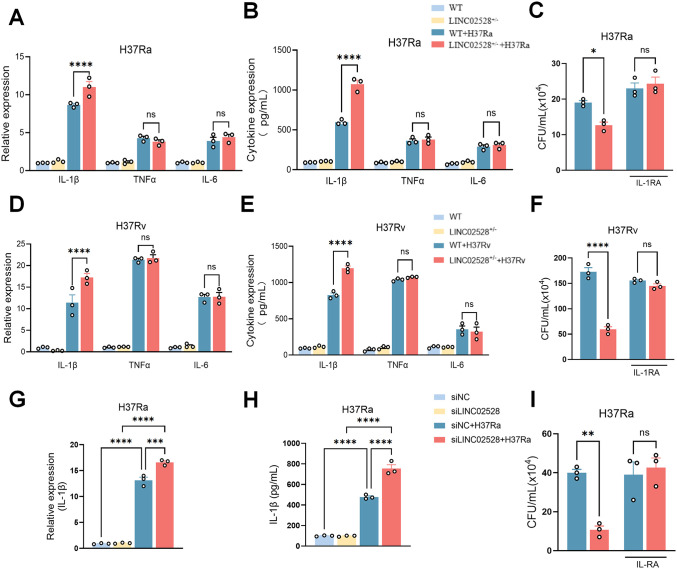
LINC02528 deficiency suppressed intracellular *Mtb* growth by enhancing IL-1β production. Detection of mRNA **(A)** and protein **(B)** expression of pro-inflammatory cytokines IL-1β, TNF-α, and IL-6 in LINC02528^+/-^ macrophages infected with H37Ra 24 **h. (C)** CFU assay results for LINC02528^+/-^ macrophages infected with H37Ra after addition of IL-1 receptor agonist (RA). Detection of mRNA **(D)** and protein **(E)** expression of cytokines IL-1β, TNF-α, and IL-6 in LINC02528^+/-^ macrophages infected with H37Rv 24 **h. (F)** CFU assay results for LINC02528^+/-^ macrophages infected with H37Rv after addition of IL-1RA. Detection of mRNA **(G)** and protein **(H)** expression of cytokines IL-1β, TNF-α, and IL-6 in SiLINC02528 macrophages infected with H37Ra 24 **h. (I)** CFU assay results for SiLINC02528 macrophages infected with H37Ra after addition of IL-1RA. The data represent the mean ± SEM from 3 independent experiments. One-way ANOVA Dunnett’s multiple comparisons test was used. Not significant (ns), *p < 0.05, ** *p* < 0.01, *** *p* < 0.005, **** *p* < 0.001.

To investigate the specific mechanism by which LINC02528 knockdown enhances the bactericidal capacity of macrophages, we systematically screened the classical bactericidal pathways of macrophages. The results demonstrated that LINC02528 deficiency does not affect cell apoptosis by Annexin/PI assay (S5A Fig); does not affect cell necrosis by LDH assay (S5B Fig); does not affect autophagy by detecting ratio of LC3-II/LC3-I (S5C Fig); does not affect total reactive oxygen species (cROS) using DCFH-DA staining (S5D Fig) at 24 hours post-infection with either the H37Ra or H37Rv strains. Transcriptomic profiling 24 hours after *Mtb* (H37Ra) infection identified a distinct pattern of differentially expressed and inflammatory-related genes in LINC02528-knockdown macrophages compared to wild-type controls (|log₂(Fold Change)| > 0.5, p-value < 0.05, S6A Fig). For further qPCR and Western validation, we focused on key cytokines IL-1β, IL-6, and TNF-α, given their critical functions in anti-mycobacterial immunity. The results revealed a significant increase in both the transcriptional and secretory levels of IL-1β in LINC02528^+/-^ macrophages, whereas other pro-inflammatory factors such as TNF-α and IL-6 remained unaffected (Fig 3A and 3B). Additionally, LINC02528^+/–^ macrophages exhibited elevated IL-1β transcriptional levels at 12 h, 24 h and 48 h post-infection with *Mtb* compared to WT macrophages (S6B Fig). IL-1β has already been reported to trigger several bactericidal mechanisms in macrophages [20,21], to address the function of the elevated IL-1β production in *Mtb* infected LINC02528^+/-^ macrophages compared to controls, we used a naturally occurring IL-1 receptor antagonist (IL-1RA), an IL-1 inhibitor that binds directly to IL-1 receptors, to specifically block IL-1β signaling. We performed this closure experiment in an IL-1RA concentration-dependent manner and showed that a relatively high concentration of IL-1RA (100 ng/mL) was able to rescue *Mtb* growth in LINC02528^+/–^ macrophages compared with WT macrophages (S6C Fig). The CFU assay in LINC02528^+/–^ macrophages with and without IL-1RA was repeated and the results are shown in Fig 3C.

To validate the key findings, we employed the highly virulent H37Rv strain (Fig 3D-3F) and siRNA-mediated LINC02528 knockdown (Fig 3G-3I) for experimentation, obtaining consistent results. LINC02528^+/–^ macrophages exhibited significantly upregulated transcriptional and translational levels of IL-1β upon H37Rv infection, while exerting minimal impact on TNF-α and IL-6 expression (Fig 3D and 3E). Moreover, compared to the attenuated H37Ra strain, H37Rv-infected macrophages demonstrated an earlier and more pronounced increase in IL-1β levels (Fig 3D and 3E). Additionally, IL-1RA effectively blocked the suppression of intracellular *Mtb* growth mediated by LINC02528^+/–^ macrophages infected with H37Rv (Fig 3F). Specifically, siRNA-mediated LINC02528 knockdown significantly enhanced IL-1β transcription and secretion in THP-1 macrophages infected with *Mtb* (Fig 3G and 3H). The addition of IL-1RA rescued the bacteria growth inhibition caused by LINC02528 deficiency (Fig 3I). Collectively, these results indicate that LINC02528 knockdown suppresses intracellular *Mtb* growth through enhanced IL-1β production.

### LINC02528 binds directly to TOMM22 to inhibit IL-1β production

**Fig 4:**
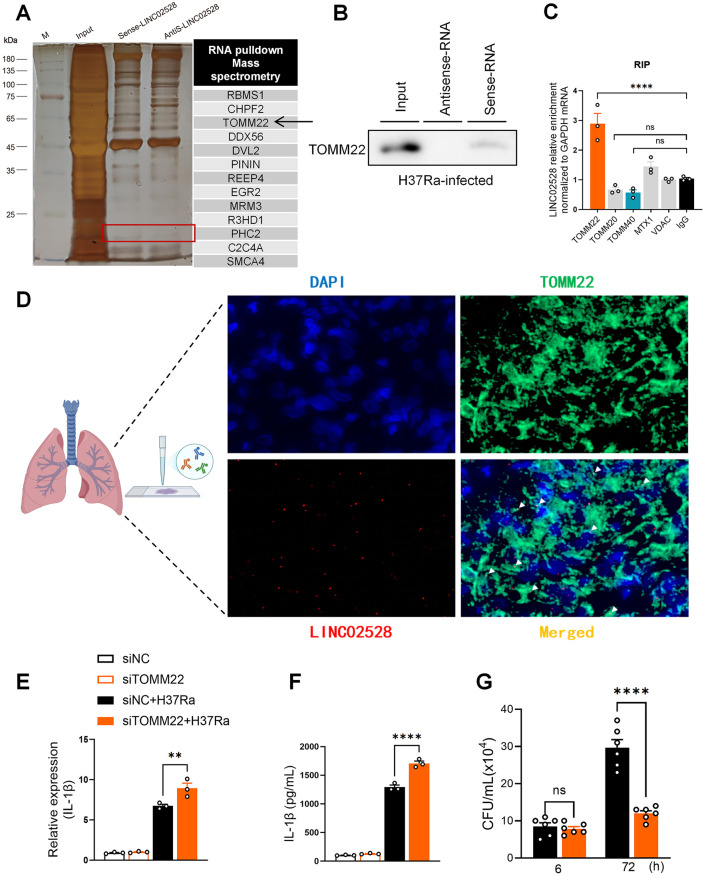
LINC02528 binds directly to TOMM22 to inhibit IL-1β production. **(A)** Silver staining and mass spectrometry of RNA pull-down assay results showing sense-LINC02528 binds to TOMM22 in THP-1 macrophages infected with H37Ra 24 **h. (B)** Immunoblotting results showing sense-LINC02528 binds to TOMM22 with same treatment above. **(C)** RNA immunoprecipitation (RIP) assay results showing TOMM22 antibody binds of LINC02528 with same treatment above. **(D)** RNAscope assay data representing the co-detection (white arrows) of LINC02528 and TOMM22 in lung tissue of a systematic TB patient (This figure was generated using BioRender (https://biorender.com/)), DAPI is blue, TOMM22 is green, LINC02528 is red dots, merged as yellow, scar bar = 2 μM. Detection of **(E)** mRNA and **(F)** protein expression of pro-inflammatory cytokines IL-1β in SiTOMM22 macrophages infected with H37Ra 24 **h. (G)** CFU assay results for SiTOMM22 macrophages infected with H37Ra (MOI = 5) for 6 and 72 **h.** The data represent the mean ± SEM from 3 independent experiments. One-way ANOVA Dunnett’s multiple comparisons test was used. Not significant (ns), *p < 0.05, ** *p *< 0.01, *** *p* < 0.005, **** *p* < 0.001.

To identify the interacting proteins of LINC02528 in regulating IL-1β production, we employed a biotinylated LINC02528 probe (sense-lncRNA) for RNA pull-down experiments to explore potential protein candidates targeted by LINC02528. Initial mass spectrometry screening identified TOMM22 among the top 13 high-confidence interactors of LINC02528 (Fig 4A). Previous analysis revealed that LINC02528 expression increased in mitochondria upon *Mtb* infection (Fig 2E), suggesting a functional link to mitochondrial proteins like TOMM22. Functional validation via siRNA knockdown in *Mtb*-infected macrophages showed that TOMM22 depletion significantly enhanced IL-1β secretion (p < 0.001), implicating its regulatory role in inflammation and supporting its selection as a key target of LINC02528. We further validated this LINC02528-TOMM22 interaction using an immunoblotting assay, confirming that the LINC02528 probe (sense-lncRNA) specifically captured endogenous TOMM22 in the whole-cell lysate of macrophages, while the LINC02528 antisense probe exhibited no binding activity to TOMM22 (Fig 4B). TOMM22, a mitochondrial outer membrane protein and one of the receptor proteins of the translocase of the outer membrane (TOM) complex [22], has been associated with limited reported functions. Additionally, protein immunoprecipitation assays demonstrated that endogenous TOMM22 specifically interacts with LINC02528, whereas other members of the TOMM family or other mitochondrial proteins exhibited no such interaction (Fig 4C). Furthermore, the RNAscope imaging assay was employed to visualize the in-situ localization of LINC02528 and TOMM22 in biopsy samples of diseased lung tissue obtained from a patient with active pulmonary tuberculosis, histopathologic features showed the presence of necrotic granulomatous inflammation of this lung tissue (S7A-D Fig). The results demonstrated distinct co-localization of LINC02528 (red) and TOMM22 (green), as indicated by white arrows (Fig 4D). Notably, LINC02528 signaling was detected in both the nucleus and cytoplasm (Figs 4D and S7A-D).

To identify which LINC02528-interacting proteins involved in IL-1β production and macrophage bactericidal activity, we performed a screening assay using gene-specific siRNAs. Top five candidates were individually knocked down in macrophages using siRNA, cells were then infected with *Mtb* (MOI = 5, 24h), and IL-1β secretion was quantified by ELISA. TOMM22 emerged as the top hit because its knockdown led to a significant increase in IL-1β production upon *Mtb* infection (p < 0.001 vs. controls), suggesting a potential regulatory role in inflammation. Remarkably, the specific knockdown of TOMM22 using siRNA significantly enhanced IL-1β production (Fig 4E and 4F) and also exhibited the ability to inhibit intracellular *Mtb* growth (Fig 4G).

**Fig 5:**
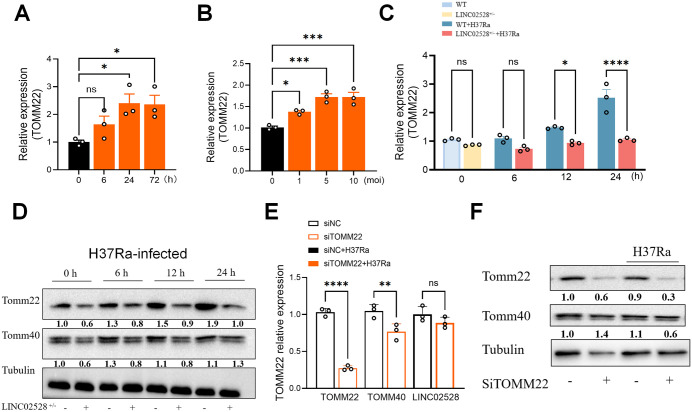
*M. tuberculosis* infection increased TOMM22 expression and acts downstream of LINC02528. mRNA levels of TOMM22 were detected in a **(A)** time and **(B)** *Mtb*-dose dependent way. **(C)** mRNA levels of TOMM22 were detected in LINC02528^+/-^ macrophages via an infected time-increased (0, 6, 12, 24 h) way. **(D)** Protein expression of TOMM22 and TOMM40 were detected in LINC02528^+/-^ macrophages by western blot in an increased infected-time way. The effect of TOMM22 silencing on **(E)** TOMM40 mRNA expression was assessed by qPCR, while **(F)** the protein level was analyzed by western blot. The data represent the mean ± SEM from 3 independent experiments. One-way ANOVA Dunnett’s multiple comparisons test was used. Not significant (ns), *p < 0.05, ** *p* < 0.01, *** *p* < 0.005, **** *p* < 0.001.

Subsequently, we analyzed the expression patterns of TOMM22 in *Mtb*-infected macrophages. The results demonstrated that TOMM22 expression progressively increased over time and with increasing infection dosage, parallelling the expression trend of LINC02528 during *Mtb* infection (Fig 5A and 5B). In contrast, in LINC02528^+/-^ and *Mtb*-infected cells, TOMM22 expression was consistently suppressed from 0 to 24 h after *Mtb* infection (Fig 5C). Regarding other related genes, TOMM40 mRNA levels showed only a slight elevation from 0 to 24 h after *Mtb* infection and were not significantly affected by the regulation of LINC02528^+/-^ as was the case for TOMM20, which was regulated by LINC02528^+/-^ to affect the expression of its mRNA levels (S6D Fig). Consistent with these mRNA findings, protein expression levels exhibited analogous trends (Fig 5D). Additionally, we employed siRNA to knockdown TOMM22, which resulted in a significant reduction in TOMM22 mRNA levels, accompanied by a slight decrease in TOMM40 mRNA levels, but did not affect LINC02528 (Fig 5E). And protein levels showed that silencing did knock downTOMM22 and TOMM40 expression was only reduced a little bit after infection (Fig 5F). These results indicate that LINC02528 acts upstream of TOMM22.

### LINC02528/TOMM22 interaction regulates IL-1β production by metabolic reprogramming

**Fig 6:**
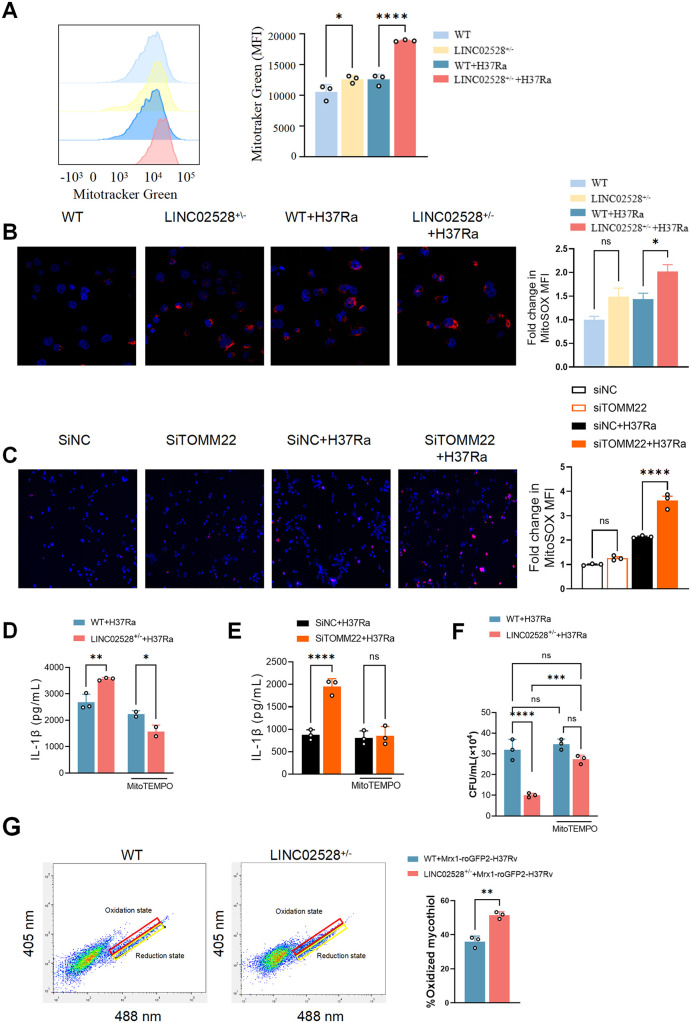
LINC02528/TOMM22 interaction affects mitochondrial mass and mtROS. **(A)** Mitochondrial content was measured with MitoTracker green; histogram shows calculated mean fluorescence intensity (MFI) data. **(B)** Mitochondrial (mt)ROS detection using MitoSOX Red mitochondrial superoxide probe at 6 h in LINC02528^+/-^ macrophages (left). Histogram analysis of MFI of mtROS by flow cytometry (FCM) (right). **(C)** mtROS assay in SiTOMM22 macrophages after infection at 6 **h.** Protein expression of pro-inflammatory cytokines IL-1β in **(D)** LINC02528^+/-^ and **(E)** SiTOMM22 macrophages infected with H37Ra and treated with mtROS inhibitor MitoTEMPO (50 μM) for 24 **h. (F)** CFU of LINC02528^+/-^ infected with H37Ra and treated with MitoTEMPO (50 μM) for 72 **h. (G)** Redox status of intracellular *Mtb* population within LIN02528 + /-macrophages. FACS dot plot (left) of infected macrophages with Mrx1-roGFP2-expressing H37Rv in WT and LINC02528^+/-^ macrophages at 24 h after infection. Bar graph (right) represents percentage of oxidized subpopulation of macrophages during *Mtb* infection. The data represent the mean ± SEM from 3 independent experiments. One-way ANOVA Dunnett’s multiple comparisons test and unpaired student’s t test were used. Not significant (ns), *p < 0.05, ** *p *< 0.01, *** *p* < 0.005, **** *p* < 0.001.

Given that TOMM22 is a critical regulatory protein for mitochondrial function, we further conducted a systematic investigation into how LINC02528/TOMM22 interaction modulates mitochondrial function, encompassing mitochondrial fission and fusion, as well as mitochondrial quality and quantity. The Transmission electron microscopy (TEM) results demonstrate that LINC02528-deficient macrophages exhibit increased mitochondrial number under both basal conditions and upon *Mtb* infection (S8A-C Fig). Furthermore, infected LINC02528-knockout cells showed a reduced mitochondrial length, consistent with the phenomenon of “pro-inflammatory mitochondrial fragmentation [23]. Flow cytometry with Mitotracker Green demonstrated that LINC02528^+/–^ increased mitochondrial mass (Fig 6A), prompting us to investigate whether it affects oxidative stress at the mitochondrial level. In addition, the results showed that LINC02528 or TOMM22 knockdown significantly increased mtROS production (Fig 6B and 6C). Importantly, blocking mtROS with MitoTEMPO (the optimal concentration was verified by cell viability and mtROS inhibition assays, S8D and S8E Fig), a specific inhibitor of mtROS, reversed the elevated IL-1β production in LINC02528^+/-^/SiTOMM22 macrophages compared to WT macrophages infected with *Mtb* (Fig 6D and 6E), and almost rescue *Mtb* growth in LINC02528^+/-^ cells (Fig 6F). For comparison, the addition of ROS scavenger NAC showed no effect on the inhibition *Mtb* growth within LINC02528^+/-^ macrophages compared to WT macrophages (S8F Fig). Direct measurement of the mycothiol redox potential in intracellular *Mtb* (using the Mrx1-roGFP2 biosensor) confirmed that LINC02528-deficient macrophages induce significant oxidative stress within the bacteria (Fig 6G). This result provides the missing causal link, demonstrating that elevated host mROS compromises bacterial redox homeostasis, leading to restricted bacterial growth.

**Fig 7:**
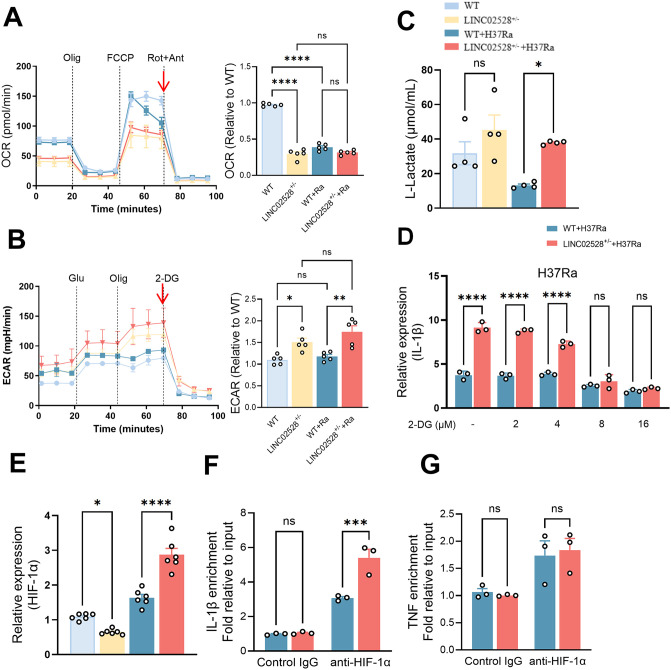
LINC02528/TOMM22 interaction regulates IL-1β production by metabolic reprogramming towards enhanced glycolysis. **(A)** Mitochondrial stress assay showing change in oxygen consumption rate (OCR) of wild type and LINC02528^+/-^ macrophages with or without *Mtb* infection for 24 **h. (B)** Glycolysis stress assay showing change in extracellular acidification rate (ECAR) of wild type and LINC02528^+/-^ macrophages with or without *Mtb* infection for 24 **h. (C)** L-Lactate assay was measured by kit (n = 4). **(D)** mRNA expression of IL-1β in LINC02528^+/-^ macrophages infected with H37Ra 24 h in a 2-DG concentration increased way. **(E)** mRNA expression of HIF-1α in wild type and LINC02528^+/-^ cells infected or not with H37Ra 24 **h.** CHIP-qPCR identification of HIF-1α as an **(F)** IL-1β- or **(G)** TNF-α promoter-binding transcription factor in LINC02528^+/-^ and *Mtb*-infected macrophages. The data represent the mean ± SEM from 3 independent experiments. One-way ANOVA Dunnett’s multiple comparisons test was used. Not significant (ns), *p < 0.05, ** *p *< 0.01, *** *p* < 0.005, **** *p* < 0.001.

Further, the seahorse assay was employed to characterize the impact of LINC02528 on mitochondrial metabolism. The results demonstrated that LINC02528^+/-^ induced a metabolic shift from oxidative phosphorylation (OXPHOS) to glycolysis (includes basal glycolysis and glycolytic capacity) in macrophages (Fig 7A and 7B). ECAR assays showed a significant increase in glycolysis by LINC02528^+/-^ after *Mtb* infection, the relative differences were calculated where the arrows point (Fig 7B, *p* < 0.001), which may explain the observed increase in mtROS. Furthermore, L-lactate measurement, which was commonly regarded as a direct indicator of glycolytic activity, revealed that LINC02528-deficiency led to elevated lactate levels (Fig 7C), further supporting the notion that macrophages inhibit further intracellular bacterial growth if prolonged glycolysis occurs. Collectively, these findings highlight LINC02528’s critical role in regulating antimicrobial response by locally inducing high concentrations of mtROS through numerically increased but low-functioning mitochondria, while metabolic reprogramming triggered by impaired mitochondrial function (e.g., enhanced glycolysis) further inhibits bacteria.

Previous studies have demonstrated that enhanced glycolysis is typically associated with upregulation of IL-1β production, thereby increasing the bactericidal activity against intracellular bacteria [11]. We further demonstrated that treatment with various concentrations of 2-Deoxy-D-glucose (2DG, an analog of glucose, a glycolytic inhibitor) abolished the increased IL-1β mRNA levels in LINC02528-deficient macrophages following *Mtb* infection (Fig 7D). Notably, hypoxia-inducible factor 1α (HIF-1α), acting as a transcription factor, modulates glycolysis-related genes and preferentially regulates IL-1β transcription [24]. We confirmed that LINC02528^+/-^ resulted an increase in HIF-1α transcriptional levels under *Mtb* infection conditions (Fig 7E). Subsequently, using chromatin immunoprecipitation (ChIP)-qPCR, we provided evidence that more HIF-1α enriched at the promoter of IL-1B in LINC02528^+/–^ macrophages compared to WT macrophages infected with *Mtb* (Fig 7F). Furthermore, HIF-1α showed no effect on TNF-α (Fig 7G). Collectively, these data demonstrate that LINC02528 regulates the intracellular growth of *Mtb* by modulating IL-1β expression through the TOM22/HIF-1α pathway.

## Discussion

Emerging evidence indicates that the metabolic-immune axis, which is influenced by host-pathogen interactions and is primarily responsible for controlling energy metabolism and protective immunological responses, is the basic biological basis of immunity against tuberculosis [25,26]. However, the precise molecular mechanisms governing this axis remain to be fully elucidated. In their quiescent state, macrophages predominantly rely on oxidative phosphorylation (OXPHOS) for energy production. However, upon stimulation by *Mtb*, Toll-like receptors (TLRs) or pro-inflammatory cytokines trigger metabolic reprogramming to meet heightened energy demands, thereby driving cellular activation and facilitating downstream effector functions [27,28]. *Mtb* in the host hijacks host defense mechanisms and manipulates host innate immune responses and cell death pathways, thereby complicating the outcome of TB infection [29].

In recent years, an increasing number of researchers have focus on the immune regulatory role of lncRNA in the pathogenesis and prognosis of TB. Existing studies have shown that changes in the expression of lncRNA may affect the macrophages abilities to phagocytose and clear pathogens, as well as to present antigens to T lymphocytes, thereby influencing the anti-tubercular immune response [8]. However, its functions and mechanisms of action have not yet been fully elucidated. Our study’s main finding was that LINC02528, which was shown to be increased in TB patients’ macrophages, is a functional lncRNA that controls IL-1β-dependent protective immunity in TB by binding specifically to TOMM22 and regulating mitochondrial activity, including mROS production and glycolysis metabolism.

External signals and the metabolic state alter mitochondrial morphology [30], and *Mtb* stimulation normally leads to mitochondrial fragmentation [31,32], but the lack of LINC02528 may have prevented further fragmentation of the mitochondria. TOMM22, a component of the multi-subunit mitochondrial TOM complex, which includes TOMM20, TOMM22, and TOMM70, plays a critical role in recognizing and facilitating the transport of mitochondrial precursor proteins. Notably, TOMM22 was identified as the sole member of the complex that directly interacts with LINC02528. These data collectively support LINC02528’s role in mitochondrial function. Besides, these TOM complex proteins’ deficiencies have been associated with neurodegenerative diseases and cardiac lesions [33]. However, most studies have focused on the structure of TOMM22, and few have investigated its biological functions. An early paper mentions that the depletion of either TOMM40 or TOMM22 reduced *Chlamydia caviae* infection of *Drosophila* cells [34]. Recently, one study revealed that TOMM22 overexpression promotes aggressive cell growth in in pancreatic cancer by modulating mitochondrial protein import and function, suggesting its potential as an early diagnostic/prognostic biomarker [35]. Our study indicates that TOMM22 promotes the intracellular growth of *Mtb*, suggesting a mechanism warranting further exploration in TB pathology.

Cytokines are major regulators of macrophage activation that fine tune macrophage responses to achieve the effective clearance of pathogens while limiting the amount of inflammation to avoid toxicity and tissue damage [36,37]. Mitochondria are critical for the signaling activity of three major innate immune pathways, including RIG-1/MAVS, NLRP3, and TLR9. MAVS might be a key sensor of mtROS that acts to promote host defense and inflammation [38]. MAVS associates with NLRP3 and promotes its oligomerization, which leads to caspase-1 activation and drives IL-1β production [39,40]. Mitochondrial DNA can also activate NLRP3 and is sensed by TLR9, which leads to immune and inflammatory gene expression [41]. Thus, future consideration should be given to studying alterations in these three innate immune signaling pathways to investigate whether LINC02528 triggers mtROS production through these pathways.

In our experiments, we did not observe a significant change in apoptosis via Annexin V/PI staining following *Mtb* infection at an MOI of 5 for 24 hours. However, a significant increase in LDH release was detected under the same conditions (S5A and S5B Fig). It is worth noting that no significant differences in either apoptosis or LDH release were found between wild-type and LINC02528^+/−^ macrophages, with or without infection. This suggests that, at this early time point, LINC02528 knockdown does not influence *Mtb*-induced cell death.

Our discovery that the lack of LINC02528 eliminated intracellular *Mtb* growth, reversed by an IL-1 receptor-antagonist, highlights LINC02528’s role in modulating host antimicrobial activity by regulating IL-1β expression. IL-1β is a myeloid-derived pro-inflammatory cytokine that orchestrates innate and adaptive immune responses to clear pathogens. Jayaraman *et al*. found that IL-1β recruits additional antimicrobial effector molecules to directly destroy *Mtb* in human and murine macrophages [20]. Through the amplification of TNF production and TNFR1 cell surface expression, IL-1β directly increases TNF signaling in macrophages, which in turn activates caspase-3. According to their findings, IL-1β stimulates macrophages’ antimicrobial immunity by controlling TNFR signaling and caspase-3 activation [20]. Active IL-1β, rather than its precursor pro-IL-1β, has been implicated in various biological mechanisms; however, the precise cellular secretion process remains unclear [42,43]. A positive correlation between mitochondrial ROS activity and the presence of active IL-1β in the supernatant of LINC02528-deficient macrophage cell lines was observed in our study. This might be because, under pathological conditions, high mtROS levels usually lead to oxidative damage and inflammation and can exacerbate tuberculosis progression, whereas mtROS inhibitors alleviate bacterial loads/severity, as shown in mice [44]. Taken together, the interplay between mtROS and IL-1β may create a positive feedback loop, where the oxidative stress induced by mtROS leads to the production of IL-1β, which in turn can lead to further immune activation and potentially more mtROS production. Further studies into these pathways can provide insights into therapeutic approaches for tuberculosis and other infectious diseases.

The balance between mitochondrial function and glycolysis may lead to protective immunity formation in TB. Gleeson *et al.* recently demonstrated the importance of glycolysis in host defenses against intracellular *Mtb*, highlighting its role in the development of tuberculosis [16]. Increased glycolysis has been suggested to promote macrophage activation by increasing levels of IL-1β, which is critical for macrophage containment of *Mtb* [45]. In addition, many reports refer to a shift in the cellular metabolic state from oxidative phosphorylation to glycolysis and an elevation in mtROS, all of which are shown to function in the effective elimination of *Mtb* [28]. This metabolic reprogramming serves two critical purposes: firstly, it rapidly generates ATP to power antimicrobial processes like phagocytosis; secondly, glycolytic intermediates provide essential biosynthetic precursors. For example, they feed into the pentose phosphate pathway to produce NADPH for oxidative burst and ribose-5-phosphate for nucleotide synthesis, facilitating effective pathogen elimination. Besides, increased glycolysis in macrophages potentiates late IL-1β transcription via HIF-1α were reported by previous other groups [40,46] and therefore, we examined (a) whether HIF-1α promotes the transcription of IL-1β by CHIP-qPCR in LINC02528-deficient macrophages during *Mtb* infection (Fig 7F and 7G), it shows HIF-1α regulates IL-1β transcription but not with TNF in this context; (b) if increased flux through glycolysis (Fig 7B) was fueling the increased pro-inflammatory IL-1β response in LINC02528-deficient and *Mtb*-infected macrophages. Blocking glycolysis with 2DG (Fig 7D) abolished the increased IL-1β mRNA levels in LINC02528-deficient macrophages after *Mtb* infection, implicating general transcriptional induction of the IL-1β gene as a key process regulated by LINC02528. This metabolic shift provides the necessary energy and metabolic intermediates for inflammatory responses; while mtROS generated during *Mtb* infection can promote this metabolic reprogramming, enhancing glycolytic activity. Increased glycolysis supports the production of pro-inflammatory cytokines, including IL-1β. However, why TNF-α expression is unaffected by LINC02528-deficiency is unclear. In summary, mtROS are typically seen as an upstream factor that influences glycolytic pathways and the production of IL-1β in *Mtb*-infected macrophages, coordinating metabolic reprogramming and inflammatory responses to enhance the host’s immune defense against the infection.

We propose LINC02528 as a checkpoint exploited by *Mtb* to suppress IL-1β-mediated immunity. Its knockdown enhances bacterial control, suggesting therapeutic potential; however, translational strategies should be phase-specific: promoting IL-1β activity early in infection while restraining it in chronic disease. Genetic profiling of IL-1β variants and LINC02528 expression could guide personalized adjunctive therapy—for instance, IL-1β blockade in severe TB or LINC02528 inhibition in drug-resistant cases. Supporting this, we previously linked excessive IL-1β responses to TB progression in humans [47]. Future work should evaluate LINC02528-targeting agents (e.g., antisense oligonucleotides) alongside inflammasome inhibitors in preclinical models to optimize efficacy and safety.

Admittedly, a major limitation of this investigation is our inability to directly prove that LINC02528 influences intracellular *Mtb* infection by binding/collaborating with TOMM22, as their double depletion leads to cell death. The study also lacked a rescue experiment to further confirm the function of LINC02528. Additionally, LINC02528 is expressed in different immune cells, but only macrophages were studied, and understanding its cell-type-specific roles will be important for future studies. Notwithstanding these limitations, this study has highlighted an important biological target for TB.

In conclusion, we identified the importance of the lncRNA LINC02528 in *Mtb* infection and described a previously unknown mechanism by which LINC02528 regulates mitochondrial metabolism and suppresses pro-inflammatory IL-1β to maintain macrophage homeostasis and promote *Mtb* growth in macrophages. LINC02528 levels inversely correlate with TB disease severity, suggesting that we may be able to develop interventions against *Mtb* using host-directed therapies targeting such lncRNA-protein interactions.

## Methods

### Ethics statement

This study was reviewed and approved by the Medical Ethics Committee of Shenzhen Baoan Hospital. The approval for this study was granted under the reference number BYL20210327. All participating individuals provided written informed consent prior to donating blood samples.

### Patients and clinical specimens

We recruited the four batches of TB patients from Shenzhen Bao’an District People’s Hospital in 2019. The batch 1 includes 62 patients with active tuberculosis (TB), 46 healthy controls (HC) and 21 patients with pneumonia (PN). We collected PBMC samples from the batch 1 for whole-transcriptome sequencing, and then performed lncRNA profiling. The batch 2 includes 28 new TB patients and 28 healthy people, we used the batch 2 to verify the RNA seq results and measure the lncRNA expression in the PBMCs. The batch 3 includes 37 untreated TB patients, 26 TB patients after 1 month of standardized anti-tuberculosis treatment, and 18 TB patients after 3 month of standardized anti-tuberculosis treatment. The batch 4 includes 8 new TB patients and 8 new healthy individuals, we used the batch 4 to collect PBMCs and further do the cell subpopulation sorting. The batch5 includes 29 healthy controls, 22 latent tuberculosis infection (LTBI), and 28 active TB patients, we used the batch5 to collect PBMCs and do the RNA extraction and qPCR analysis.

The diagnostic criteria for tuberculosis were referred to the “Diagnosing Tuberculosis” reported by Clinical and Laboratory Diagnosis for Tuberculosis in 2024 [48]. Healthy control enrolled subjects had normal chest imaging and no history of TB exposure; Pneumonia patients (community-acquired, caused by a virus or bacteria, CT suggestive of pneumonia) enrolled cases had not received radiation, chemotherapy and other immunotherapy and had no history of TB exposure. All subjects were excluded from patients with comorbid hypertension, diabetes mellitus, chronic obstructive pulmonary disease (COPD), autoimmune diseases, and immunosuppressive drugs; patients with HBV, HCV, HIV infection and/or AIDS were excluded; and pregnant or lactating women were excluded. There was no statistically significant difference in age and gender between patients with active tuberculosis and the control group.

### Bacterial strains and growth condition

Mycobacterial strains, including BCG, the attenuated *M. tuberculosis* strain H37Ra, and the virulent strain H37Rv, were cultured at 37°C in Middlebrook 7H9 broth supplemented with 0.2% (vol/vol) glycerol, 0.25% (vol/vol) Tween-80, and 10% OADC, under shaking conditions. Following this incubation period, bacteria reached the mid-logarithmic growth phase, with an OD600 of 0.6, and were then ready for infection. For the CFU assay using solid media, the bacteria were grown on Middlebrook 7H10 plates supplemented with 0.5% glycerol, 1 g/L L-asparagine, and 10% OADC. H37Rv expressing Mrx1-roGFP2 (*Mtb*-roGFP2) was cultured under the same conditions as other *Mtb* strains, with the exception of the addition of 50 µg/mL hygromycin to the medium, which was required to maintain the biosensor plasmid. Heat-killed H37Ra (HK-H37Ra) and heat-killed BCG (HK-BCG) were used in this study under experimental conditions of 65°C for 30 minutes. Laboratory strains of *Escherichia coli* (*E.coli*) and *Staphylococcus aureus* (*S.aureus*) were used. Both strains were routinely cultured in Luria-Bertani (LB) broth or on LB agar plates.

### Cell culture

PBMC isolation, as well as hMDMs and THP-1 macrophages differentiation, were performed as previously described [49]. Briefly, after obtaining PBMCs from whole blood via density gradient centrifugation (2000 rpm, 20 min) in Ficoll-Paque medium, the cells were incubated overnight to allow adhesion. The adherent monocytes were differentiated into hMDMs by treatment with 40 ng/mL of human macrophage colony-stimulating factor (PeproTech) for 5–6 days. Human monocytic THP-1 cells were plated at 5 × 10^5^ cells/mL in 12-well plates (Costar) and treated with 40 ng/mL PMA for 36–48 hours until they differentiated into macrophages. Both hMDMs and THP-1 macrophages were maintained in fresh prewarmed media supplemented with L-glutamine (2 mM) and 10% fetal bovine serum at 37°C until further use.

### Macrophage infection model

THP-1 macrophages were infected with H37Ra or H37Rv *Mtb* in a time- (0-6-24-72 h) and concentration- (multiplicity of infection [MOI]=0,1,3,5) dependent manner, then washed twice with 1 × PBS. Subsequently, total cell lysates, precipitated supernatants, and pellets of cells at different stages were analyzed by RT-qPCR, immunoblotting, ELISA, etc.

### Transcriptome sequencing and analysis

PBMCs (TB batch1) were isolated from whole blood and used to perform transcriptome sequencing. Total RNA was extracted by using TRIzol; Library preparation is performed using Optimal Dual-mode mRNA Library Prep Kit (BGI-Shenzhen, China). A certain amount of RNA treated with Hieff NGS MaxUp Human rRNA Depletion Kit (Yeasen-Shanghai, China) to remove rRNA. After reacting at a suitable temperature for a fixed time period, RNAs are fragmented with fragmentation reagents. Then First-strand cDNA is generated using random hexamer-primed reverse transcription, followed by a second-strand cDNA synthesis in a new reaction system containing dUTP. The synthesized double strand cDNA is subject to end repairment reaction. After cDNA end repairment, a single‘A’nucleotide is added to the 3’ ends of the blunt fragments through A tailing reaction. Then the reaction system for adaptor ligation is configured to ligate adaptors with the cDNAs, and finally, the library products are amplified through PCR reaction and subjected to quality control. Next, the single-stranded library products are produced via denaturation. The reaction system for circularization is set up to get the single-stranded circularized DNA products. Any single stranded linear DNA molecules will be digested. The final single strand circularized library is amplified with phi29 and rolling circle amplification (RCA) to make DNA nanoball (DNB) which carries more than 300 copies of the initial single stranded circularized library molecule. The DNBs are loaded into the patterned nanoarray and sequencing reads of PE 100/150 bases length (each sample requires at least 100 million reads) are generated on G400/T7/T10 platform (BGI-Shenzhen, China). Sequencing reads were aligned to the reference genome using HISAT2. Transcripts were assembled with StringTie, and novel lncRNAs were identified by filtering for length (>200 nt) and low coding potential using tools like CPC2. Differentially expressed genes (DEGs) were identified, with the filtering thresholds set at an adjusted p-value ≤0.05 and |log2 fold change (FC) | ≥ 1. The Database for Annotation, Visualization and Integrated Discovery (DAVID) was exploited to perform Reactome enrichment analysis based on the mRNAs significantly correlated to LINC02528. Enriched Reactome pathways were determined according to the cut-off criterion of an adjusted P-value ≤ 0.05.

### CFU assay

THP-1 macrophages were infected with *Mtb* H37Ra (MOI = 5) for 6 hours or H37Rv (MOI = 5) for 4 hours, then washed with 1 × PBS. The cells were incubated in fresh RPMI 1640 medium for a further 72 hours. Subsequently, the cells were lysed with 0.1% SDS, and serial dilutions were plated onto 7H10 plates. The numbers of colony-forming units (CFU) were counted after incubation at 37°C for 2–3 weeks, as previously described [49].

### Generation of LINC02528-knockout THP-1 cell clone by CRISPR/Cas9

To efficiently deplete endogenous LINC02528 expression in a THP-1 cell line, we constructed lentiviral LINC02528-targeting gRNA using LentiCRISPRv2 (one vector system: pGL3-U6 gRNA vector). The LINC02528-targeting gRNA sequences were picked from a list generated by the online Single-guide RNA Design Tool (Broad Institute, Cambridge, MA, USA) and inserted into a LentiCRISPRv2 plasmid. Lentivirus production and THP-1 transduction were performed as previously described [50]. The following gRNA coding sequences were used to generate lentiviruses targeting the indicated genes for inactivation: gRNA-B1: TGGAGGATTTGGATAAGTGGAG; gRNA-B2: TCCTAACCTCTAGTACCTTAGGG. After selection with 2 μg/ml puromycin and 3 μg/ml blasticidin in culture medium for 1 week, single-cell colonies were cultured and verified for genome deletion by PCR and qPCR.

### In vitro transcription and LINC02528 RNA-protein pull-down assay

For transcription in vitro, biotin-labeled sense (F, forward) or anti-sense (R, reverse) RNAs were synthesized using the T7 RNA Polymerase transcription kit and biotin-labeled rNTPs mix. Then 3 μg of isolated biotin-labeled RNA was heated to 90°C for 2 minutes to disrupt the secondary structure and placed on ice for 2 minutes for secondary structure formation. At least 1 × 10^7^ H37Ra-infected THP-1 cells were lysed in NT2 (+) buffer (50 mM Tris-HCl pH 7.0, 150 mM NaCl, 1 mM MgCl2, 0.05% NP-40, 1 mM PMSF, 10 mM ribonucleoside vanadyl complex) supplemented with propidium iodide (PI) and 1 U/μL SUPERase-In, followed by sonication and centrifugation to separate the cell lysate. Then the lysate was precleared with 50 μL streptavidin-coupled beads by rotating at 4°C for 30 minutes. Folded RNA was added to the precleared cell lysate and rotated at room temperature for 1 hour before 20 μL washed streptavidin-coupled beads were added to the reaction and rotated at 4°C for 1 hour, followed by four washes using the NT2 (+) buffer supplemented with 1 mM PMSF and 10 mM ribonucleoside vanadyl complex. Proteins captured by the beads were separated by SDS-PAGE, followed by their identification with silver staining and mass spectrometry (described in S1 Text). The precipitated RNA-protein complexes were also analyzed by western blotting.

### RNA immunoprecipitation: reverse pull-down assay

For RNA immunoprecipitation (RIP), approximately 5 × 10^6^ THP-1 cells with formaldehyde crosslinking were harvested and lysed with Cell Lysis Buffer supplemented with PI and 1U/μL SUPERase-In. Then the mixture was sonicated and centrifuged to obtain a clear cell lysate. Next, 3 μg TOMM22 monoclonal antibody conjugated with Pierce Protein A/G Beads were added to the clarified lysate and hybridized for 4 hours at 4°C. Other antibodies (3 μg) were also used as controls: mouse anti-IgG, mouse monoclonal anti-TOMM20, rabbit polyclonal anti-TOMM40, rabbit polyclonal anti-MTX1, and rabbit polyclonal anti-VDAC1. After five washes in NT2 (+) buffer, the RNA and proteins captured on the beads were isolated using TRIzol according to the manufacturer’s instructions. RNA fractions were subjected to qPCR analysis.

### RNAscope

The RNAscope assay was performed using the RNAscope Multiplex Fluorescent Reagent Kit v2 (Advanced Cell Diagnostics, 323100) with modifications to the standard protocol. Briefly, formalin-fixed paraffin-embedded necrotic lung tissue sections (5 μm) from a TB patient (56-year-old male, batch1) were subjected to target retrieval by boiling for 10 min, followed by protease IV treatment at 40 °C for 7 min. Custom-designed probes targeting LINC02528 isoform 1 were hybridized and amplified according to the kit instructions. RNA signals were detected using TSA Vivid Fluorophore 570 (1:1500 dilution; Advanced Cell Diagnostics) incubated at 40 °C for 30 min. For simultaneous RNA and protein detection, following the TSA amplification step, sections were incubated with primary antibodies against CD68 (1:200 dilution, Abcam) or TOMM22 (1:200 dilution, invitrogen) overnight at 4 °C, followed by incubation with HRP-conjugated secondary antibodies (488-conjugated). Tyramide signal amplification was applied for protein visualization. RNAscope 3-plex Positive and Negative Control Probes were included in each experiment. Whole-slide imaging was performed using a Keyence BZ-810 fluorescence microscope under a × 40 objective.

### Chromatin immunoprecipitation

THP-1 cells were seeded into 100-mm dishes and treated as described above. Cells were treated with 1% formaldehyde for 10 minutes at 37°C and rinsed twice with ice-cold PBS supplemented with phenyl-methanesulfonyl fluoride and protease inhibitors (Roche). Cells were then lysed, sonicated on ice, and centrifuged. Aliquots of the supernatants were heated to break the crosslinks and recover genomic DNA for an input control. The experiments were carried out according to the kit protocol (One-Day Chromatin Immunoprecipitation Kit, EZ-Magna ChIP A/G, Catalog # 17–10086, Millipore), and the final purified DNA samples were used for qPCR. Primers targeting the IL-1β and TNF-α promoter regions were shown in S1 Table.

### Subcellular fractionation, RNA extraction and RT-qPCR

THP-1 cells were harvested before and after *Mtb* infection for subcellular fractionation. The nuclear and cytoplasmic fraction were separated using a hypotonic lysis buffer followed by centrifugation. The mitochondria fractionation was isolated using a Cell Mitochondrial Separation Kit (C3601, Beyotime) according to the manufacturer’s instructions. The purify of each fraction was confirmed by Western blot analysis using specific markers: Lamin B1 for the nucleus, β-Actin for the cytoplasm, VDAC1 for mitochondria, and GAPDH as a cytosolic marker. Quality control was performed by Western blotting on the protein collected from each fraction.

Total RNA was extracted from each fraction using the RNeasy kit (Omega) according to the manufacturer’s instructions. Contaminating DNA was removed and reverse transcription was performed using HiScript II Q RT SuperMix for qPCR (Vazyme, China). Target gene expression was analyzed with the SYBR Green Real-Time PCR Master Mix (Bimake, USA) on the 7500 Fast real-time PCR system (Applied Biosystems, ThermoFisher, USA). The relative target genes’ mRNA expression was normalized to reference gene (GAPDH) and calculated using the 2^–ΔΔCT^ method. All primer sequences are listed in S1 Table.

### Western blotting

Cells were washed with PBS and lysed on ice using radio immunoprecipitation assay lysis buffer supplemented with a protease inhibitor cocktail. The protein concentration was determined using a BCA protein kit; 20 μg cell lysates were loaded and separated using SDS-PAGE and then transferred onto PVDF membranes (Millipore). Blocking was carried out using PBS with Tween 20 containing 5% BSA for 1 hour at room temperature. The membranes were incubated overnight at 4°C with the primary antibodies. They were then incubated with HRP-conjugated secondary antibodies for 1 hour at room temperature and visualized using ECL detection solution (ThermoFisher). Digital images of the protein bands were acquired using the Chemiluminescence Image System (Minichem, China).

### ELISA

Supernatants from cell culture were assayed for human IL-1β, TNF-α, and IL-6 with ELISA kits according to the manufacturer’s instructions.

### OCR and ECAR assay

The THP-1 macrophages were seeded at 8 × 10^4^ per well, differentiated, and infected with or without H37Ra for 24 hours, as previously described [51]. We used the Seahorse XF Cell Mito Stress Test kit (103015–100; Seahorse Bioscience) for the OCR assay. Electron transport chain inhibitors were added every 20 min from the beginning in the following order: oligomycin (1.5 mM), FCCP (1 mM), and then a combination of rotenone (0.5 mM) and antimycin A (0.5 mM). For the ECAR assay, the Seahorse XF Glycolysis Stress Test kit (103020–100; Seahorse Bioscience) was used. All readings were normalized to the protein level of each well using a bicinchoninic acid (BCA) protein assay kit (P0011; Beyotime). Data were determined using the Seahorse XFe24 extracellular flux analyzer (Agilent Technologies, Santa Clara, CA, USA).

### mtROS and cROS detection

Differentiated THP-1 macrophages were treated as described above for 6 hours and washed three times in pre-warmed serum-free RPMI 1640 medium. Endogenous cytoplasmic ROS (cROS) and mitochondrial ROS (mtROS) levels were determined by incubating the cells with 10 μM 2,7-diacetate dichlorofluorescein (H2DCF-DA; Invitrogen) and 5 μM MitoSOX Red Mitochondrial Superoxide Indicator, respectively, for 10 minutes at 37°C in the dark. After incubation, ROS levels were determined by flow cytometric measurement of the MFI using a FACSAria II flow cytometer, and data were analyzed with FlowJo software version 10. MitoSOX Red staining of mtROS was also observed by flow cytometric measurement or confocal microscopy, and images were obtained using an Olympus FV1000 confocal microscope (Nikon A1R).

### Redox profiling of intracellular *Mtb*-roGFP2

To assess the mycothiol redox state of *M. tuberculosis* ex vivo, we used an H37Rv strain expressing the Mrx1-roGFP2 biosensor. Control and LINC02528-knockdown THP-1 cells were infected with the bacteria at an MOI of 5. Twenty-four hours post-infection, cells were washed, treated with 10 mM NEM for 5 minutes, and fixed with 4% PFA. Flow cytometry was performed on a BD FACS Aria Fusion, measuring the 510 nm emission ratio upon excitation at 405 nm and 488 nm. Data were analyzed with FlowJo v10 (BD Biosciences), with gating applied according to an established strategy [52,53].

### Statistical analysis

All statistical analyses were performed with GraphPad Prism Version 9.4.1 (GraphPad Software Inc.). Unpaired two-tailed Student’s t test was used to analyze the difference between two groups, and one-way analysis of variance (ANOVA) was used to compare differences among multiple groups. Data are expressed as the mean ± SEM. Differences were considered statistically significant when *P* < 0.05.

## Supporting information

S1 Fig (related to Fig 1). LINC02528 is upregulated in TB patients. (A) Venn diagram of differentially expressed lncRNAs shared among the three groups from RNA-seq data generated in PBMCs from TB patients (n = 62), HC (n = 46), and PN patients (n = 26). (B) Volcano plots showing differentially expressed lncRNAs in HC vs TB and PN vs TB. (C) Enriched Reactome pathways related to LINC02528. (D) FPKM values of screened top four lncRNAs among TB, PN and HC groups. (E) CFU assays in SiFAM225A and SiLINC02555 macrophages infected with H37Ra 6 and 72 h*.* The data represent the mean±SEM from 3 independent experiments. One-way ANOVA Sidak’s multiple comparisons test was used. Not significant (ns), *p < 0.05, ** *p* < 0.01, *** *p* < 0.005, **** *p* < 0.001.
(TIFF)

S2 Fig (related to Fig 2). *M. Tuberculosis* H37Rv infection increased LINC02528 expression in vitro. (A, B) LINC02528 expression over time in THP-1 cells infected with various multiplicity of infection [MOI] values of *Mtb* H37Rv. (C) Schematic representation (icons were created with BioRender.com) of Smart silencer, a mixed oligonucleotide kit containing 3 antisense oligonucleotides (ASOs) and 3 small-interfering (si)RNAs used to knockdown LINC02528 expression. Histogram showing relative expression of LINC02528 in THP-1 macrophages transfected with each single siRNA or mixed one. The data represent the mean±SEM from 3 independent experiments. One-way ANOVA Dunnett’s multiple comparisons test was used. Not significant (ns), *p < 0.05, ** *p* < 0.01, *** *p* < 0.005, **** *p* < 0.001.
(TIFF)

S3 Fig (related to Figs 1 and 2). Gene expression of LINC02528 in latent TB subjects and subcellular fraction verification. (A) LINC02528 expression via qPCR in a cohort comprising healthy controls (HC, n = 29), LTBI individuals (n = 22), and active TB patients (n = 28). (B) Nuclear protein and cytosolic protein separation purity verified by western blot in untreated and *Mtb* infected THP-1 cells. (C) Mitochondrial protein and cytosolic protein separation purity verified by western blot in untreated THP-1 cells (icons were created with BioRender.com). The data represent the mean±SEM from 3 independent experiments. One-way ANOVA Sidak’s multiple comparisons test were used. Not significant (ns), *p < 0.05, ** *p* < 0.01, *** *p* < 0.005, **** *p* < 0.001.
(TIFF)

S4 Fig (related to Fig 2). Details of CRISPR/Cas9 based-knockout of LINC02528. (A) Schematic diagram of CRISPR/Cas9 system-mediated LINC02528 gene editing in THP-1 cell line. (B) Gel electrophoresis results showing genome-sized bands for LINC02528 mutant and wild type (WT) as control. (C) Base calling.
(TIFF)

S5 Fig (related to Fig 3). LINC02528+ ^/-^ suppresses intracellular *Mtb* growth in a manner independent of apoptosis, necroptosis, cROS and autophagy, related to Fig 3. (A)FCM analysis of Annexin/PI was detected in wild type and LINC02528+ ^**/-**^ cells with and without *Mtb* infection (H37Ra and H37Rv, MOI = 5, 24h). (B) Lactate dehydrogenase (LDH) activity in supernatants of uninfected vs. *Mtb*-infected macrophages, measured by absorbance at 490 nm. (C) Autophagy analysis marked by ratio of LC3B-II/I protein level were detected in the same experimental treatment groups. (D) Cytoplasmic (c)ROS was measured by using H2DCF-DA probes. The data represent the mean ± SEM from 3 independent experiments. One-way ANOVA Dunnett’s multiple comparisons test was used. Not significant (ns), *p < 0.05, ** *p* < 0.01, *** *p* < 0.005, **** *p* < 0.001.
(TIFF)

S6 Fig (related to Fig 3). Effects of LINC02528 deficiency on IL-1β production and bacterial burden during *Mtb* infection. (A)Heatmap analysis of differential expressed genes. Hierarchical clustering was performed on significantly differentially expressed genes (p < 0.05, |log2FC| > 0.5) and predefined inflammation-related genes between the SiLINC02528_Ra and SiNC_Ra groups. Expression values were normalized using the Z-score method. Sample groups were color-coded (SiLINC02528_Ra: pink, SiNC_Ra: blue), and gene types are annotated (Inflammation-related: green, Other-differential: purple). (B) IL-1β secretion levels in supernatants of LINC02528+ ^**/-**^ macrophages infected with H37Ra for 12, 24, and 48 hours. (C) CFU of H37Ra in LINC02528+ ^**/-**^ macrophages treated with increasing concentrations of IL-1RA (0, 10, 100 ng/mL). (D) mRNA levels of TOMM40 were detected in LINC02528+ ^/-^ macrophages via an infected time-increased (0, 6, 12, 24 h) way. The data represent the mean ± SEM from 3 independent experiments. One-way ANOVA Sidak’s multiple comparisons test was used. Not significant (ns), *p < 0.05, ** *p* < 0.01, *** *p* < 0.005, **** *p* < 0.001.
(TIFF)

S7 Fig (related to Fig 4). In situ expression of LINC02528 in granulomas of lung tissue from an active TB patient. (A)Hematoxylin and eosin staining (2 mm) of a TB patient lung tissue. (B) RNAscope was performed with the same lung tissue (Continuous slicing) to detect LINC02528, CD68 and TOMM22 probes. Red indicates the accumulation of LINC02528 in both of cell nucleus and cytoplasm. (C) CD68 (green) was detected in the macrophage, where LINC02528 was not expressed. (D) White arrows represent colocalization of LINC02528 and TOMM22 (Green) in the same cells. Scale bar = 10 µm and 2 mm. Blue fluorescence indicates nuclei (DAPI). Representative data from serial sections (5 µm) of lung tissue from a patient with typical tuberculosis are shown. The data represent the mean ± SEM from 3 independent experiments. One-way ANOVA Sidak’s multiple comparisons test was used. Not significant (ns), *p < 0.05, ** *p* < 0.01, *** *p* < 0.005, **** *p* < 0.001.
(TIFF)

S8 Fig (related to Fig 6). LINC02528+ ^/-^ not only increases mitochondrial fragmentation but also affects mitochondrial oxidative stress status. (A) Representative transmission electron microscopy (TEM) images of mitochondria in THP-1 macrophages. Images show cells from control (non-targeting) and LINC02528 knockdown (LINC02528+ ^/-^) groups, both uninfected and infected with *Mtb*. Scale bar: 2 μm. Mitochondria are outlined in yellow. Red arrows indicate intracellular *Mtb* in infected cells. Lipid droplets are marked in blue. (B) Quantification of the number of mitochondria per complete cell (*n* = 5 complete cells per group). (C) Quantification of mitochondrial area. Measurements were performed on 60–100 mitochondria per group, derived from the 5 complete cells analyzed in (B). (D) FCM analysis of mtROS inhibition by MitoTEMPO (250, 100, 50 μM). (E) Cell viability was assessed in uninfected and *Mtb*-infected macrophages following 24-hour treatment with varying concentrations of MitoTEMPO (500, 250, 100, 50, 25, 10 μM). (F) CFU assays in wild type and LINC02528+ ^**/-**^ macrophages infected with *Mtb* after treatment with NAC (5 mM). The data represent the mean ± SEM from 3 independent experiments, one-way ANOVA Dunnett’s multiple comparisons test was used. *p < 0.05, ** *p* < 0.01, *** *p* < 0.005, **** *p* < 0.001.
(TIFF)

S1 Text Supplementary methods contain detailed experimental procedures. (DOCX)

S1 Table Primers used for qPCR analysis. (XLSX)

S2 Table List of antibodies and other key reagents used in this study. (XLSX)

S1 Data Source data for gel electrophoresis and western blots. (PDF)

S2 Data Original lncRNA sequencing data in Fig 1A. (XLSX)

S3 Data Original data from the RNA-protein pull-down assay analyzed via LC-MS corresponding to Fig 4A. (XLSX)

S4 Data Original RNA-seq data used to generate S6A Fig. (XLSX)
