# Supplementary figures and images for: Mycobacterium tuberculosis manipulates LINC02528 in macrophages to modulate anti-tuberculosis metabolic immunity
Source: PLoS Pathog. 2025 Dec 23;21(12):e1013810. doi: 10.1371/journal.ppat.1013810 (PMC12768373; doi:10.1371/journal.ppat.1013810)

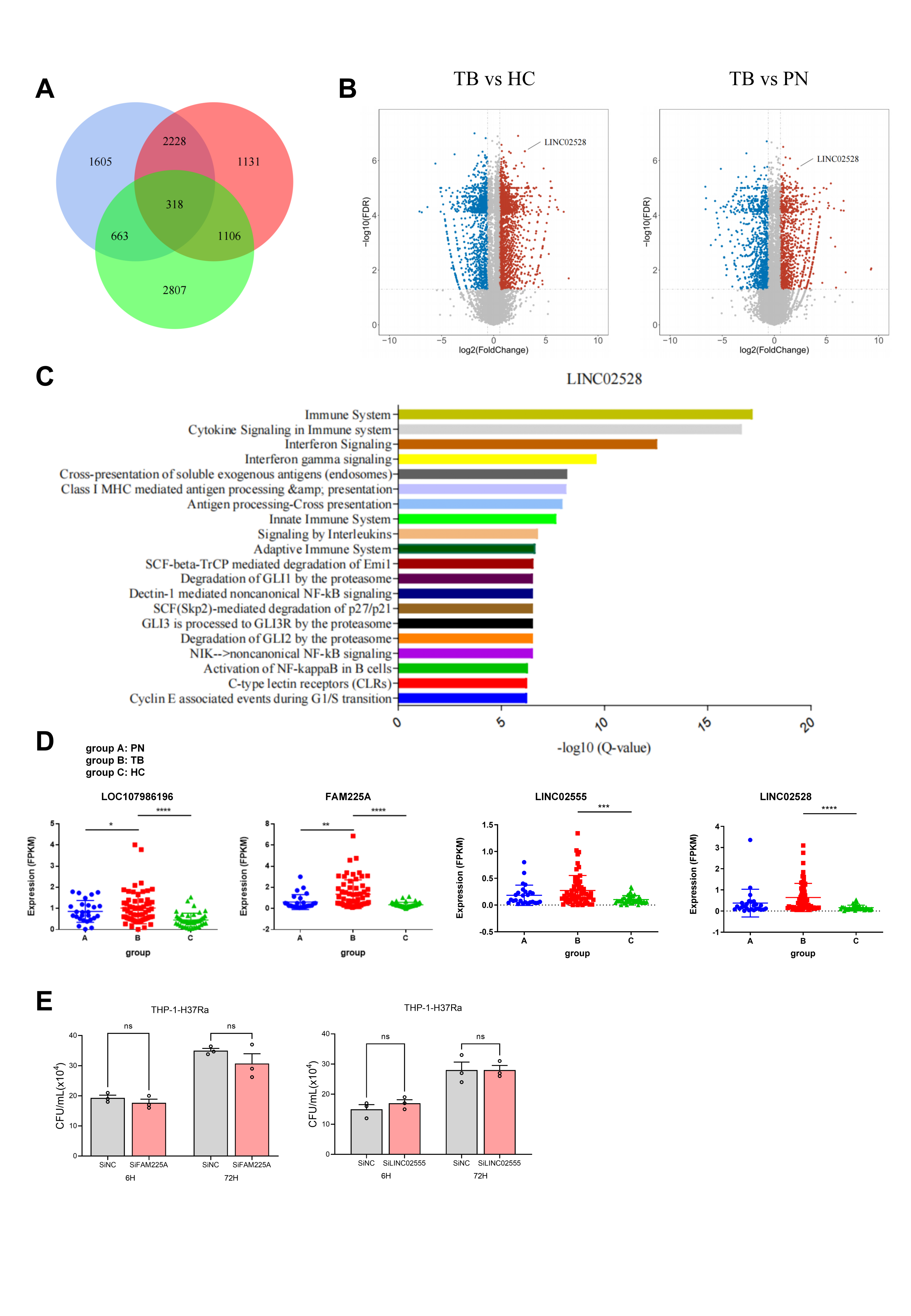

Supplement: S1 Fig — (A) Venn diagram of differentially expressed lncRNAs shared among the three groups from RNA-seq data generated in PBMCs from TB patients (n = 62), HC (n = 46), and PN patients (n = 26). (B) Volcano plots showing differentially expressed lncRNAs in HC vs TB and PN vs TB. (C) Enriched Reactome pathways related to LINC02528. (D) FPKM values of screened top four lncRNAs among TB, PN and HC groups. (E) CFU assays in SiFAM225A and SiLINC02555 macrophages infected with H37Ra 6 and 72 h. The data represent the mean±SEM from 3 independent experiments. One-way ANOVA Sidak’s multiple comparisons test was used. Not significant (ns), *p < 0.05, ** p < 0.01, *** p < 0.005, **** p < 0.001. (TIFF) [file ppat.1013810.s001.tiff]

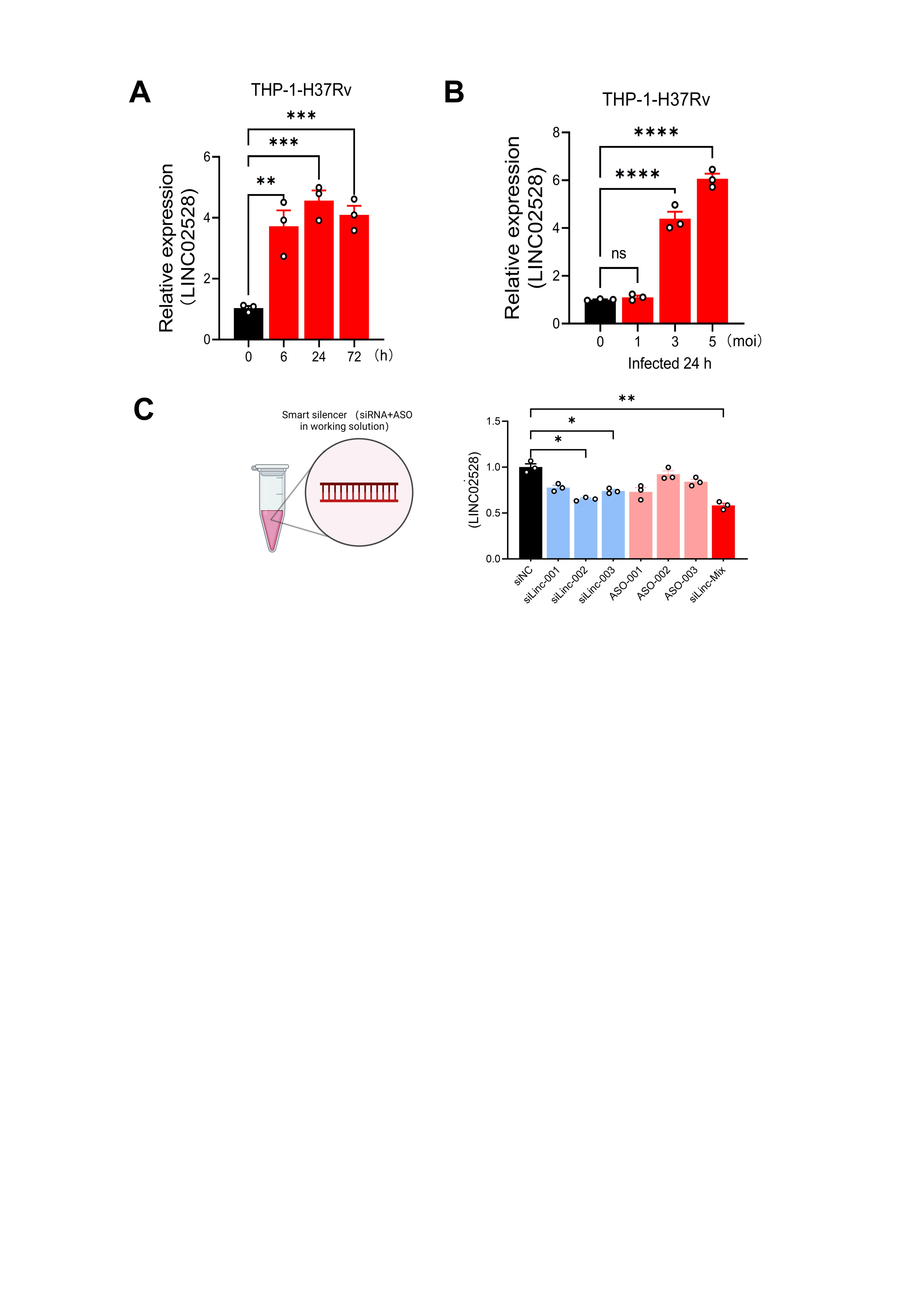

Supplement: S2 Fig — (A, B) LINC02528 expression over time in THP-1 cells infected with various multiplicity of infection [MOI] values of Mtb H37Rv. (C) Schematic representation (icons were created with BioRender.com) of Smart silencer, a mixed oligonucleotide kit containing 3 antisense oligonucleotides (ASOs) and 3 small-interfering (si)RNAs used to knockdown LINC02528 expression. Histogram showing relative expression of LINC02528 in THP-1 macrophages transfected with each single siRNA or mixed one. The data represent the mean±SEM from 3 independent experiments. One-way ANOVA Dunnett’s multiple comparisons test was used. Not significant (ns), *p < 0.05, ** p < 0.01, *** p < 0.005, **** p < 0.001. (TIFF) [file ppat.1013810.s002.tiff]

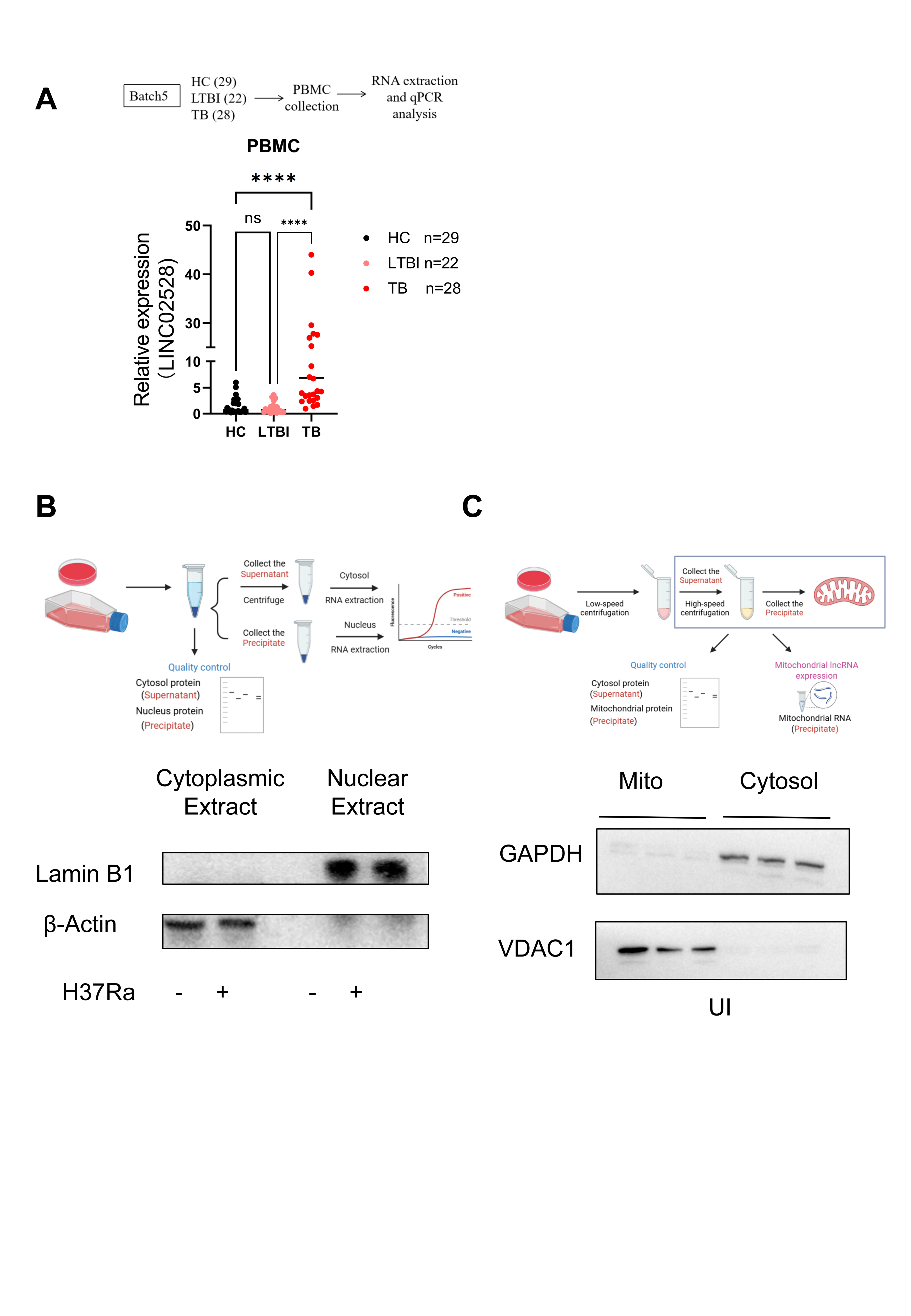

Supplement: S3 Fig — (A) LINC02528 expression via qPCR in a cohort comprising healthy controls (HC, n = 29), LTBI individuals (n = 22), and active TB patients (n = 28). (B) Nuclear protein and cytosolic protein separation purity verified by western blot in untreated and Mtb infected THP-1 cells. (C) Mitochondrial protein and cytosolic protein separation purity verified by western blot in untreated THP-1 cells (icons were created with BioRender.com). The data represent the mean±SEM from 3 independent experiments. One-way ANOVA Sidak’s multiple comparisons test were used. Not significant (ns), *p < 0.05, ** p < 0.01, *** p < 0.005, **** p < 0.001. (TIFF) [file ppat.1013810.s003.tiff]

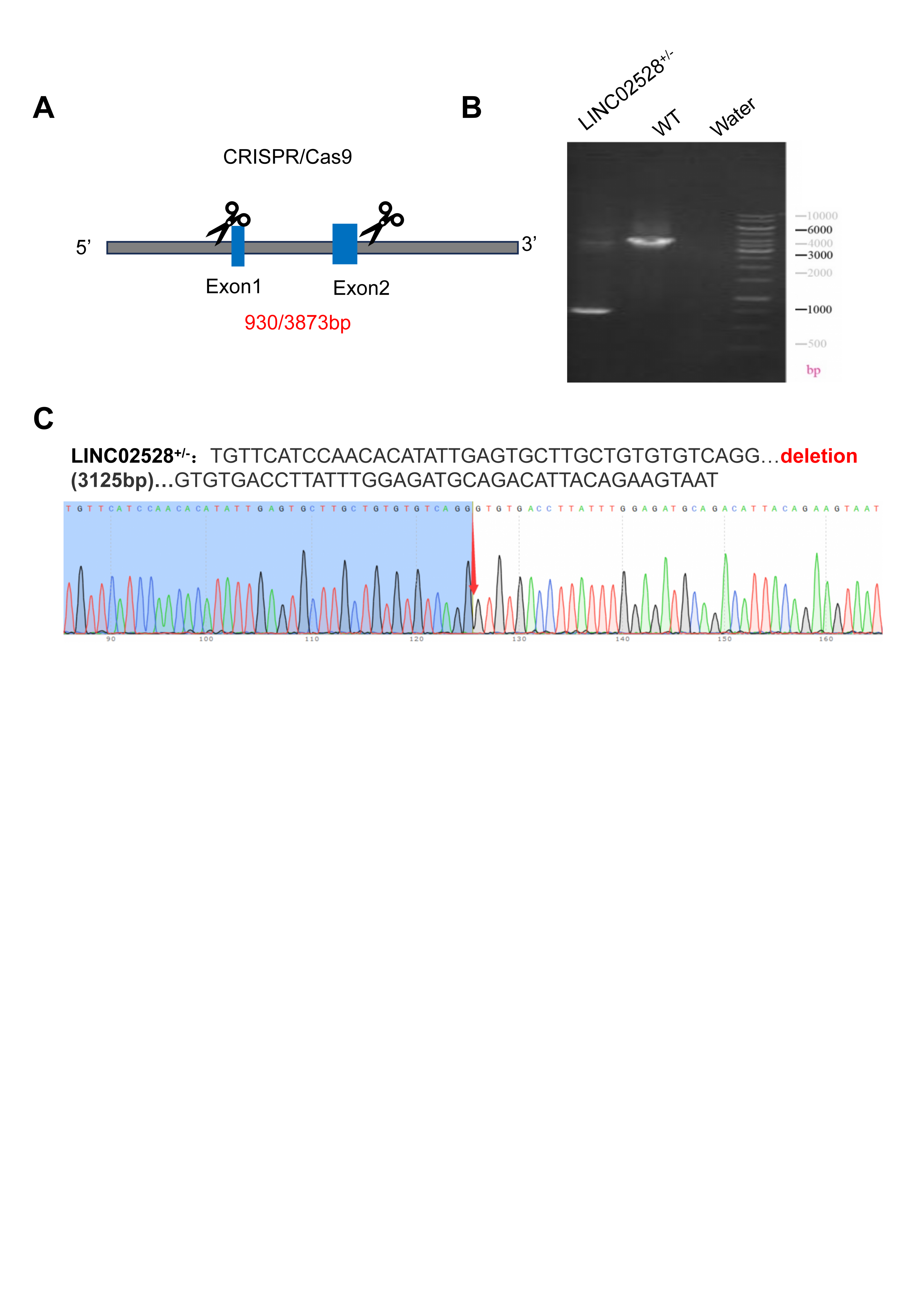

Supplement: S4 Fig — (A) Schematic diagram of CRISPR/Cas9 system-mediated LINC02528 gene editing in THP-1 cell line. (B) Gel electrophoresis results showing genome-sized bands for LINC02528 mutant and wild type (WT) as control. (C) Base calling. (TIFF) [file ppat.1013810.s004.tiff]

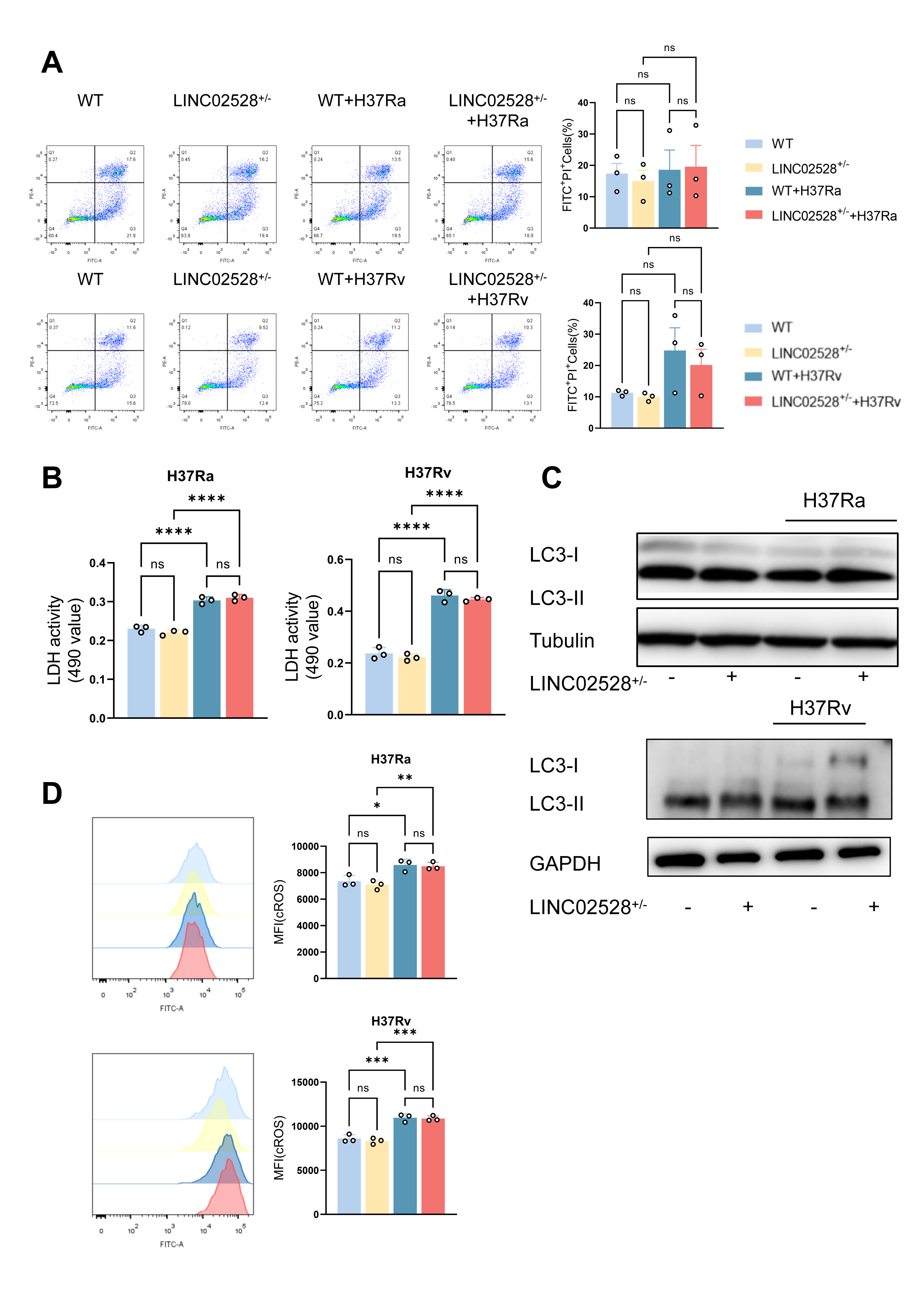

Supplement: S5 Fig — (A)FCM analysis of Annexin/PI was detected in wild type and LINC02528+ /- cells with and without Mtb infection (H37Ra and H37Rv, MOI = 5, 24h). (B) Lactate dehydrogenase (LDH) activity in supernatants of uninfected vs. Mtb-infected macrophages, measured by absorbance at 490 nm. (C) Autophagy analysis marked by ratio of LC3B-II/I protein level were detected in the same experimental treatment groups. (D) Cytoplasmic (c)ROS was measured by using H2DCF-DA probes. The data represent the mean ± SEM from 3 independent experiments. One-way ANOVA Dunnett’s multiple comparisons test was used. Not significant (ns), *p < 0.05, ** p < 0.01, *** p < 0.005, **** p < 0.001. (TIFF) [file ppat.1013810.s005.tiff]

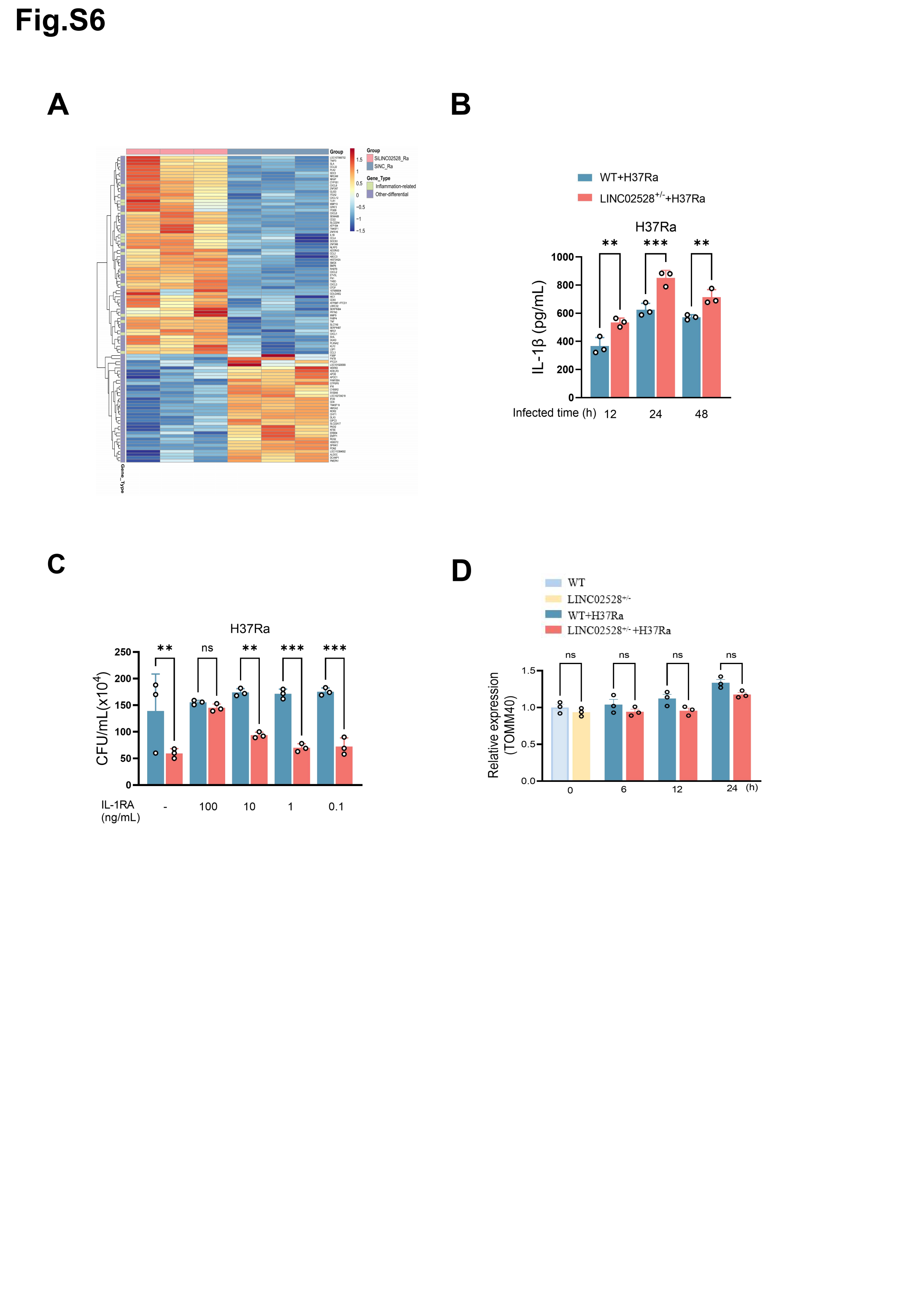

Supplement: S6 Fig — (A)Heatmap analysis of differential expressed genes. Hierarchical clustering was performed on significantly differentially expressed genes (p < 0.05, |log2FC| > 0.5) and predefined inflammation-related genes between the SiLINC02528_Ra and SiNC_Ra groups. Expression values were normalized using the Z-score method. Sample groups were color-coded (SiLINC02528_Ra: pink, SiNC_Ra: blue), and gene types are annotated (Inflammation-related: green, Other-differential: purple). (B) IL-1β secretion levels in supernatants of LINC02528+ /- macrophages infected with H37Ra for 12, 24, and 48 hours. (C) CFU of H37Ra in LINC02528+ /- macrophages treated with increasing concentrations of IL-1RA (0, 10, 100 ng/mL). (D) mRNA levels of TOMM40 were detected in LINC02528+ /- macrophages via an infected time-increased (0, 6, 12, 24 h) way. The data represent the mean ± SEM from 3 independent experiments. One-way ANOVA Sidak’s multiple comparisons test was used. Not significant (ns), *p < 0.05, ** p < 0.01, *** p < 0.005, **** p < 0.001. (TIFF) [file ppat.1013810.s006.tiff]

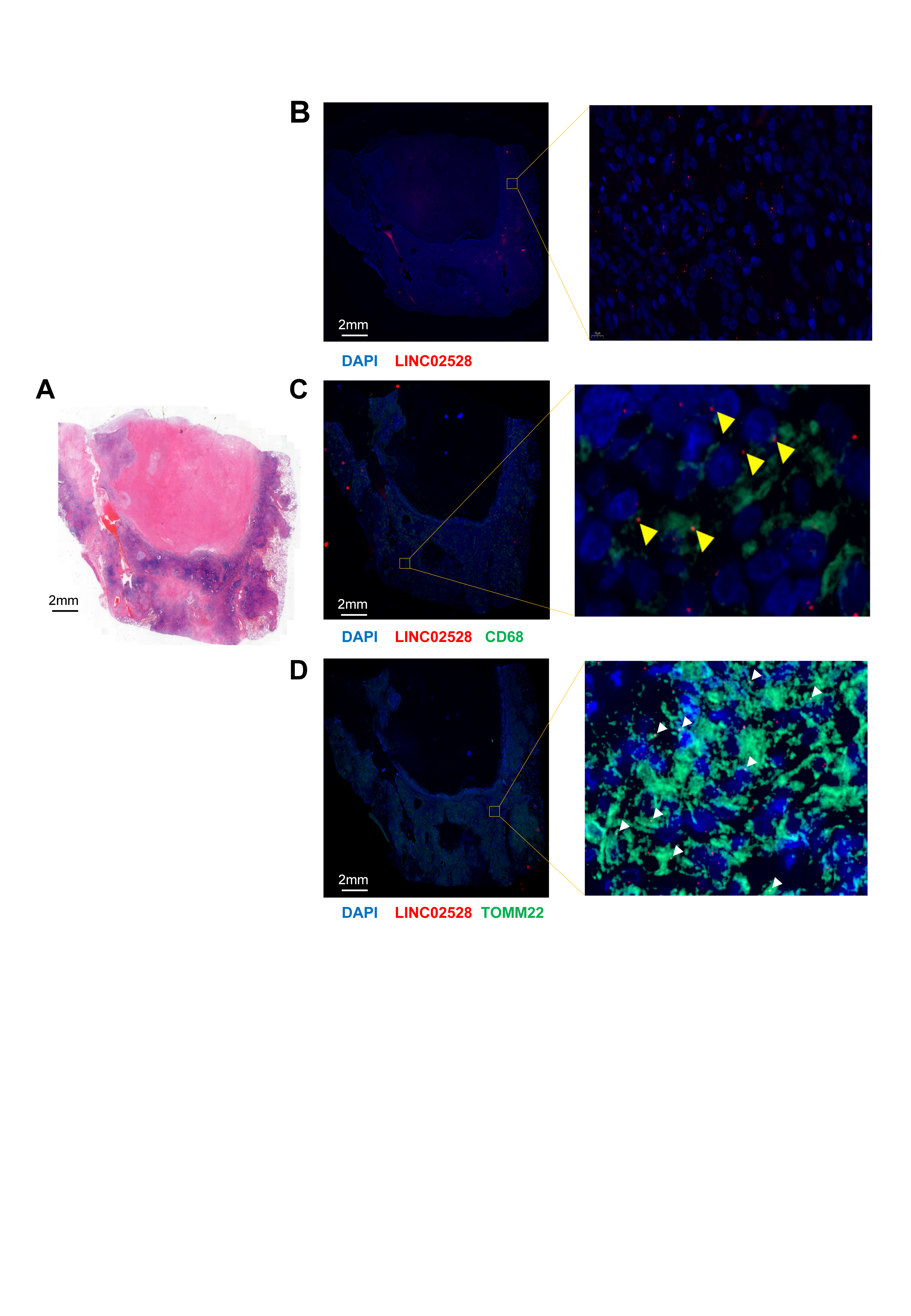

Supplement: S7 Fig — (A)Hematoxylin and eosin staining (2 mm) of a TB patient lung tissue. (B) RNAscope was performed with the same lung tissue (Continuous slicing) to detect LINC02528, CD68 and TOMM22 probes. Red indicates the accumulation of LINC02528 in both of cell nucleus and cytoplasm. (C) CD68 (green) was detected in the macrophage, where LINC02528 was not expressed. (D) White arrows represent colocalization of LINC02528 and TOMM22 (Green) in the same cells. Scale bar = 10 µm and 2 mm. Blue fluorescence indicates nuclei (DAPI). Representative data from serial sections (5 µm) of lung tissue from a patient with typical tuberculosis are shown. The data represent the mean ± SEM from 3 independent experiments. One-way ANOVA Sidak’s multiple comparisons test was used. Not significant (ns), *p < 0.05, ** p < 0.01, *** p < 0.005, **** p < 0.001. (TIFF) [file ppat.1013810.s007.tiff]

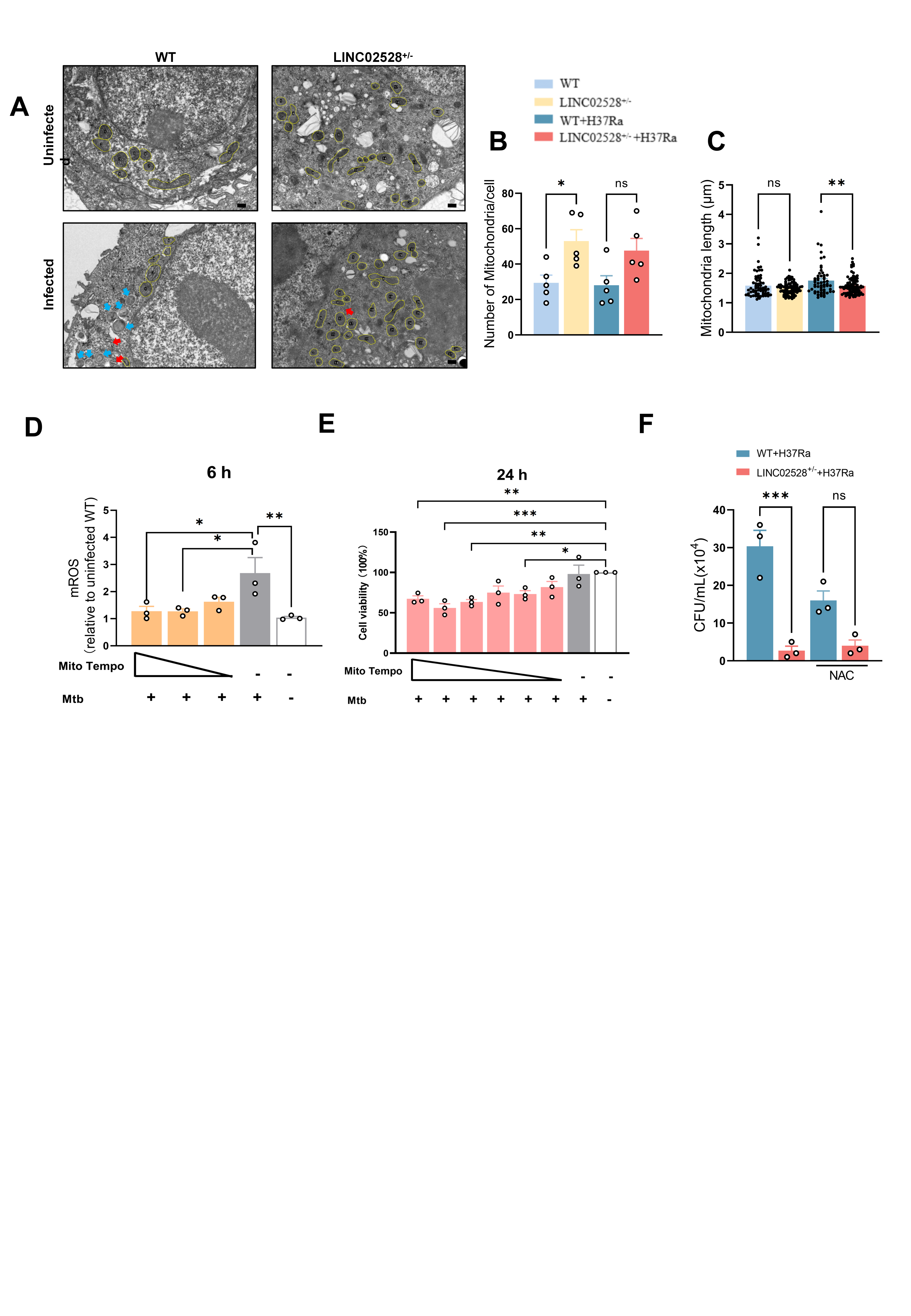

Supplement: S8 Fig — (A) Representative transmission electron microscopy (TEM) images of mitochondria in THP-1 macrophages. Images show cells from control (non-targeting) and LINC02528 knockdown (LINC02528+ /-) groups, both uninfected and infected with Mtb. Scale bar: 2 μm. Mitochondria are outlined in yellow. Red arrows indicate intracellular Mtb in infected cells. Lipid droplets are marked in blue. (B) Quantification of the number of mitochondria per complete cell (*n* = 5 complete cells per group). (C) Quantification of mitochondrial area. Measurements were performed on 60–100 mitochondria per group, derived from the 5 complete cells analyzed in (B). (D) FCM analysis of mtROS inhibition by MitoTEMPO (250, 100, 50 μM). (E) Cell viability was assessed in uninfected and Mtb-infected macrophages following 24-hour treatment with varying concentrations of MitoTEMPO (500, 250, 100, 50, 25, 10 μM). (F) CFU assays in wild type and LINC02528+ /- macrophages infected with Mtb after treatment with NAC (5 mM). The data represent the mean ± SEM from 3 independent experiments, one-way ANOVA Dunnett’s multiple comparisons test was used. *p < 0.05, ** p < 0.01, *** p < 0.005, **** p < 0.001. (TIFF) [file ppat.1013810.s008.tiff]
