# Supplementary material for: Mycobacterium tuberculosis manipulates LINC02528 in macrophages to modulate anti-tuberculosis metabolic immunity
Source: PLoS Pathog. 2025 Dec 23;21(12):e1013810. doi: 10.1371/journal.ppat.1013810 (PMC12768373; doi:10.1371/journal.ppat.1013810)
Supplement: S1 Text — (DOCX) [file ppat.1013810.s009.docx]

**Supplemental information**

***Mycobacterium tuberculosis* manipulates LINC02528 in macrophages to modulate anti-tuberculosis metabolic immunity**

Yuzhong Xu^*1,2^, Kehong Zhang^*1,2^, Sinan Li^2^, Lin Qiao^2^, Siwei Mo^3^, Wenfei Wang^4^, Jialou Zhu^5^, Xiaoqian Liu^6^, Ningjian Cai^2,9^, Chenyan Shi^2^, Yi Cai^2^, Yunlong Hu^#2^，Xinchun Chen^#2^

**SUPPLEMENTAL METHODS**

**Macrophages transfection with Smart silencer or siRNA**

Cells were transfected with target-gene-specific siRNAs, such as TOMM22 (RiboBio; stB0012489A) and TOMM40 (RiboBio; stB0003105A), using Lipofectamine RNAiMAX (Invitrogen), according to the manufacturer’s protocol, as previously described [1]. Scrambled siRNA (siNC) was used as a negative control. After transfection for 48 hours, the efficiency of knockdown was determined by qRT-PCR.

For IncRNA silencing, we used the Ribo^TM^ lncRNA Smart Silencer for human LINC02528/LINC02555/FAM225A, which is a pool containing three siRNA and three antisense oligonucleotides:

| LINC02528 | CCATATGCAAACCCATGTA  GAAACCTAGAGAAACAGAT  GAGAAACAGATGCCCAGAT  CTCCGCAGAGATAACCAGAT  CTACCGAGAGACCTCCAAAC  CACTCAACAAACCTACAATG |
| --- | --- |
| LINC02555 | CCGCAAGAAGTAAGTTGAA  CCCGAAGTCACAAAGATAA  GCATGCTACTTATAACACT  TCTCTGTGATCTGGCCACAG  CTGAAACAACATAGTGCAGG  AAGCAGCACTATGGAGCCAT |
| FAM225A | GAGCTAATTCCGTGCCGATG  TGTGACTGAGTACCTTGGGA  ATCTCTGCCCTGAGTATTGA  GGTTGGTGCTGACCTCAAA  GGATGTCCACCACTCTCCA  CACCTCCTACATGTTTATA |

**RNA seq analysis**

RNA sequencing was performed on LINC02528-knockdown (SiLINC02528_Ra) and control (SiNC_Ra) THP-1 macrophages 24 hours post-infection with *M. tuberculosis* H37Ra (MOI=5). Analysis identified differentially expressed genes (DEGs) based on a threshold of |log₂(fold change)| > 0.5 and an adjusted p-value < 0.05. A heatmap of Z-score normalized values was plotted for these DEGs and predefined inflammation-related genes to visualize expression patterns.

**LC-MS/MS analysis**

The lyophilized peptide fractions were re-suspended in 2% acetonitrile containing 0.1% formic acid, and 2ul aliquots of which was loaded into a nanoViper C18 (3 μm, 100 Å) trap column. The online Chromatography seperation was performed on the Easy nLC 1000 system (ThermoFisher). The trapping, desalting procedure were carried out at a volumn of 3 μL for 5 min with 100% solvent A (water/acetonitrile/formic acid (98/2/0.1%; B, 2/98/0.1%)). Then, an elution gradient of 8-38% solvent B in 30 min was used on an analytical column (50μm×15 cm C18-3 μm 100 Å). IDA (information-dependent acquisition) mass spectrum techniques were used to acquire tandem MS data on a ThermoFisher Q Exactive mass spectrometer (ThermoFisher, USA) fitted with a Nano Flex ion source. Data was acquired using an ion spray voltage of 1.9 kV, and an interface heater temperature of 275℃. The MS was operated with FULL-MS scans. For IDA, survey scans were acquired in 250 ms and up to 20 product ion scans (50 ms) were collected. Only spectra with a charge state of 2-4 were seleceted for fragmentaion by higher-energy collision energy. Dynamic exclusion was set for 25 s.The MS/MS data were analyzed for protein identiﬁcation and quantiﬁcation using PEAKS Studio 8.5. The local false discovery rate at PSM was 1.0% after searching against Human database with a maximum of two missed cleavages.The following settings were selected: Oxidation (M), Acetylation (Protein N-term), Deamidation (NQ), Pyro-glu from E, Pyro-glu from Q for variable modifications as well as fixed Carbamidomethylation of cysteine. Precursor and fragment mass tolerance were set to 10 ppm and 0.05 Da, respectively.

**Apoptosis assay**

Differentiated THP-1 macrophages were treated as described above, and collected by centrifugation at 1,000 × *g* for 5 minutes at room temperature. The cells were then resuspended in 195 µL Annexin V-FITC and 5 µL Annexin V-FITC and incubated on ice for 10 to 20 minutes in the dark. After staining with 10 µL propidium iodide, cell apoptosis was analyzed by FACSAria II flow cytometer.

**Cell viability assay**

Differentiated THP-1 macrophages were seeded in 96 well plates at 2 × 10^4^ per well and treated with different concentrations (500, 250, 100, 50, 25, 10 μM) of MitoTEMPO (Cat# SML0737) to uninfected and *Mtb*-infected macrophages for 24 h. The Cell Counting Kit-8 (CCK-8, MCE) assay has been used in the assessment of cell viability, according to the instruction manual.

**LDH assay**

To assess cytotoxicity, THP-1-derived macrophages were seeded in 96-well plates at a density of 1×10^5 cells per well and treated according to the experimental design. After a 24-hour incubation period, the cell culture supernatant was collected. The release of lactate dehydrogenase (LDH) into the supernatant, an indicator of cell membrane damage and cytotoxicity, was then quantified using a commercial LDH Assay Kit (Omega) according to the manufacturer's instructions.

**Mitochondrial Morphology Examination by Transmission Electron Microscopy (TEM)**

Wide type or LINC02528-depleted THP-1 differentiated macrophages (1 × 10^7^ cells/ml) were plated onto big dishes (10 cm diameter), then incubated with or without H37Ra (MOI = 5) for a further 24 hours at 37°C. Macrophages were collected and washed twice in PBS, then fixed with 2.5% glutaraldehyde in 0.1 M sodium cacodylate buffer (pH 7.4) for 2 hours at 4°C. Following fixation, cells were washed three times with 0.1 M sodium cacodylate buffer to remove excess fixative. Cells were post-fixed in 1% osmium tetroxide in 0.1 M sodium cacodylate buffer for 1 hour at room temperature. Samples were then dehydrated through a graded series of ethanol (50%, 70%, 90%, and 100%) for 10 minutes each, followed by two changes of 100% acetone for 10 minutes each. Dehydrated samples were infiltrated with a mixture of acetone and epoxy resin (1:1) for 1 hour, followed by pure epoxy resin overnight at room temperature. The next day, samples were embedded in fresh epoxy resin and polymerized at 60°C for 48 hours. Ultrathin sections (70–90 nm) were cut using a diamond knife on an ultramicrotome (e.g., Leica Ultracut). Sections were collected on copper grids and stained with uranyl acetate and lead citrate to enhance contrast. Stained sections were examined using a transmission electron microscope (e.g., JEOL JEM-1400) operated at 80 kV. Images were captured at various magnifications to visualize mitochondrial morphology (e.g., cristae structure, matrix density, and overall shape). The analysis was performed following the methodology described as reported [2].

**Reference**

[1] Dai Y, Zhu C, Xiao W, Huang K, Wang X, Shi C, et al. Mycobacterium tuberculosis hijacks host TRIM21- and NCOA4-dependent ferritinophagy to enhance intracellular growth. J Clin Invest. 2023;133.

[2] Lam J, Katti P, Biete M, Mungai M, AshShareef S, Neikirk K, et al. A Universal Approach to Analyzing Transmission Electron Microscopy with ImageJ. Cells. 2021;10.
