# Supplementary material for: Mycobacterium tuberculosis manipulates LINC02528 in macrophages to modulate anti-tuberculosis metabolic immunity
Source: PLoS Pathog. 2025 Dec 23;21(12):e1013810. doi: 10.1371/journal.ppat.1013810 (PMC12768373; doi:10.1371/journal.ppat.1013810)
Supplement: S1 Data — (PDF) [file ppat.1013810.s012.pdf]

**Source data 1 (Fig 4B):**

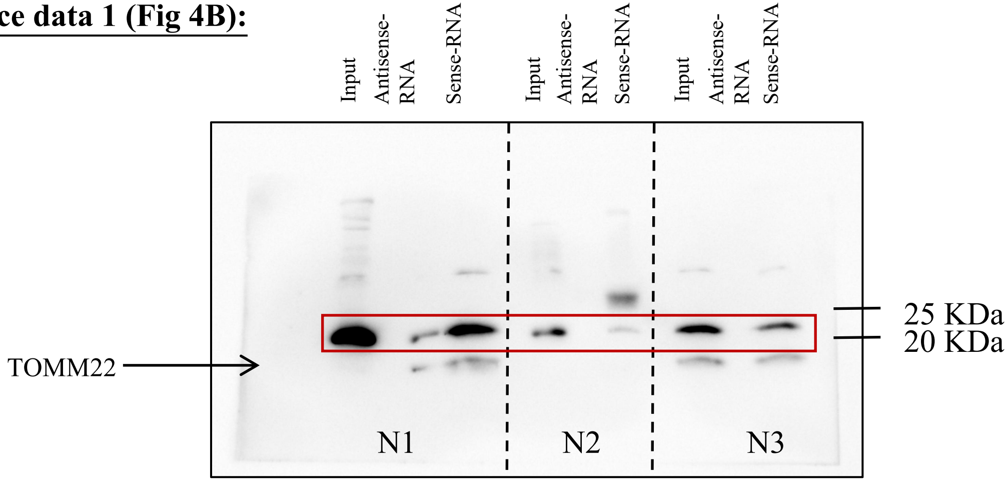


Three replicate western blot images showed that sense-LINC02528 binds to TOMM22 as a result of RNA pull-down experiments 24 hours after H37Ra infection of THP-1 macrophages.

**Source data 2 (Fig 5D):**

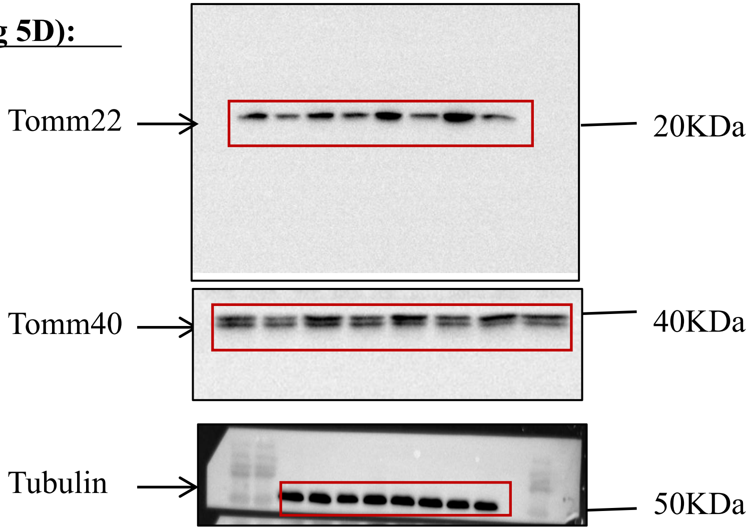


The original western blot images in Figure 5D show that protein expression of TOMM22 and TOMM40 was detected in LINC02528$^{+/-}$ macrophages in an increased infected-time way.

**Source data 3 (Fig 5F):**

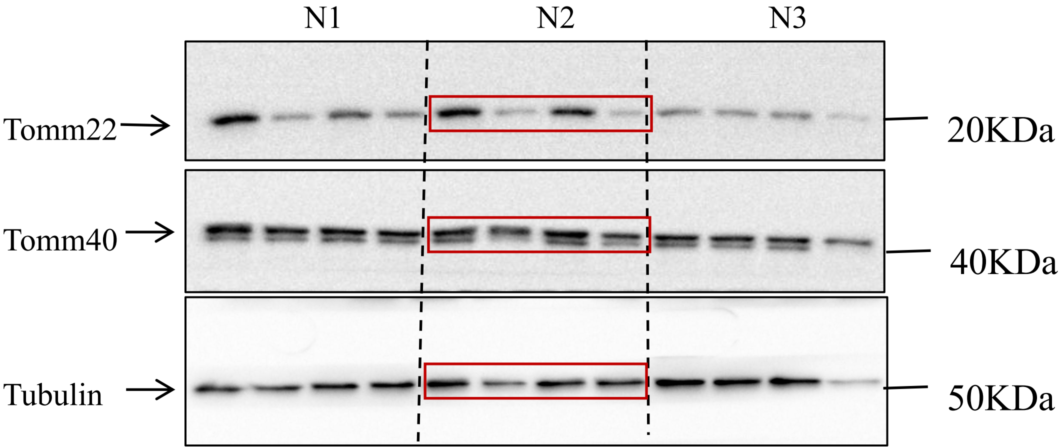


Three replicate western blot images in Figure 5F showed that protein expression of TOMM22 and TOMM40 was detected in silenced TOMM22 macrophages before and after H37Ra infection.

**Source data 4 (S3B, C Fig):**

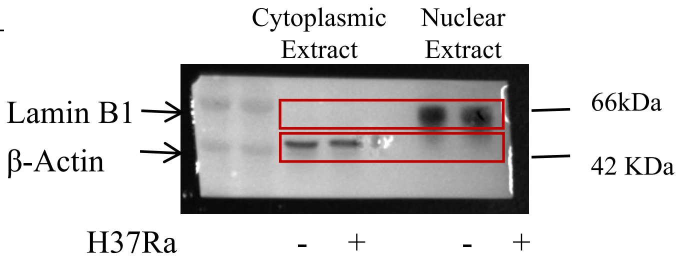


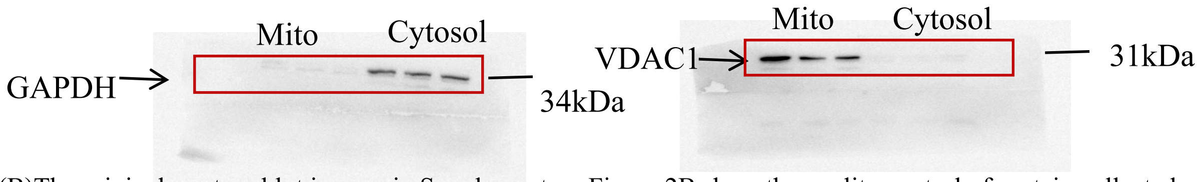


(B)The original western blot images in Supplementary Figure 2B show the quality control of protein collected form the cytoplasmic and nuclear fractions, using Lamin B1 as a nuclear marker and β-Actin as a cytoplasmic marker; (C) the original western blot images in Supplementary Figure 2C collected from the cytoplasmic and mitochondrial fractions, using VDAC1 as a mitochondrial marker and GAPDH as a cytoplasmic marker.

## Source data 5 (S4B Fig):

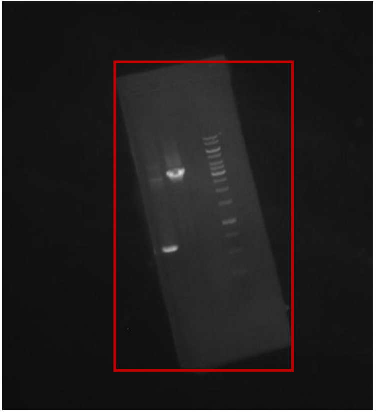

The original gel images in Supplementary Figure 3B show genome-sized bands for the LINC02528 mutant and WT and water as controls.

**Source data 6 (S5C Fig-H37Ra):**

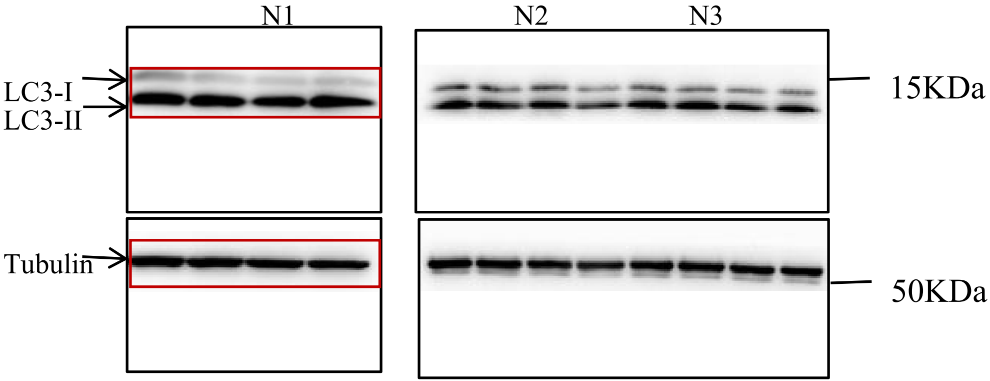


Three replicate western blot images in Supplementary Figure 5C showed that LC3B-II/I protein level in wild type and LINC02528$^{+/-}$cells upon infection with *Mtb* and without *Mtb* (H37Ra).

**Source data 6 (S5C Fig-H37Rv):**

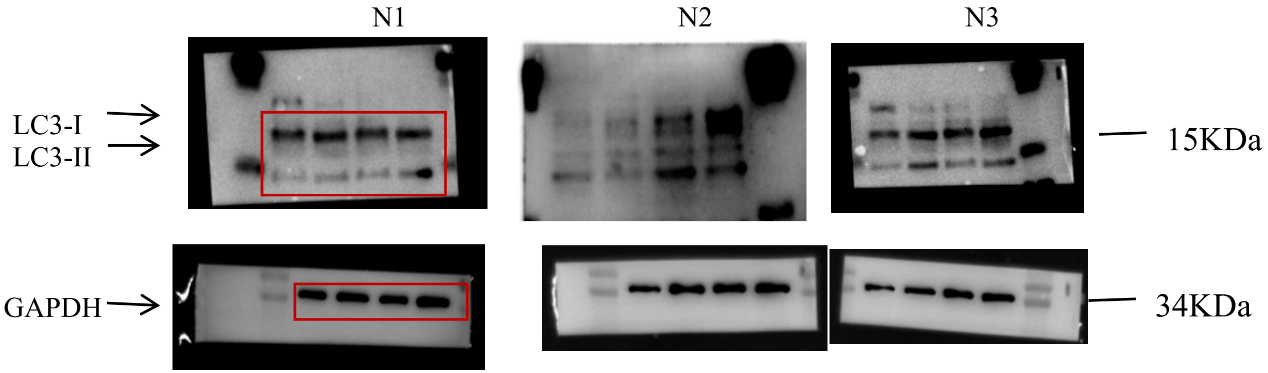


Three replicate western blot images in Supplementary Figure 5C showed that LC3B-II/I protein level in wild type and LINC02528$^{+/-}$cells upon infection with *Mtb* and without *Mtb* (H37Rv).
